# RNA helicase DDX5 modulates sorafenib sensitivity in hepatocellular carcinoma via the Wnt/β-catenin–ferroptosis axis

**DOI:** 10.1038/s41419-023-06302-0

**Published:** 2023-11-30

**Authors:** Zhili Li, Claude Caron de Fromentel, Woojun Kim, Wen-Hung Wang, Jiazeng Sun, Bingyu Yan, Sagar Utturkar, Nadia Atallah Lanman, Bennett D. Elzey, Yoon Yeo, Hao Zhang, Majid Kazemian, Massimo Levrero, Ourania Andrisani

**Affiliations:** 1 Department of Basic Medical Sciences, Purdue University, West Lafayette, IN USA; 2 Purdue Institute for Cancer Research, West Lafayette, IN USA; 3 Cancer Research Center of Lyon (CRCL) - INSERM U1052, CNRS5286, University Lyon, Université Claude Bernard Lyon 1, F69000 Lyon, France; 4 Department of Industrial and Physical Pharmacy, Purdue University, West Lafayette, IN USA; 5 Department of Biochemistry, Purdue University, West Lafayette, IN USA; 6 Department of Comparative Pathobiology, Purdue University, West Lafayette, IN USA; 7 Jiangsu Key Laboratory of Bioactive Natural Product Research and State Key Laboratory of Natural Medicines, School of Traditional Chinese Pharmacy, China Pharmaceutical University, Nanjing, China; 8 Department of Computer Science, Purdue University, West Lafayette, IN 47907 USA; 9 Hospices Civils de Lyon, Service d’Hépatologie et Gastroentérologie, Groupement Hospitalier Lyon Nord, Lyon, France

**Keywords:** Cancer, Diseases

## Abstract

Reduced expression of the RNA helicase DDX5 associated with increased hepatocellular carcinoma (HCC) tumor grade and poor patient survival following treatment with sorafenib. While immunotherapy is the first-line treatment for HCC, sorafenib and other multi-tyrosine kinase inhibitors (mTKIs) are widely used when immunotherapy is contra-indicated or fails. Herein, we elucidate the role of DDX5 in sensitizing HCC to sorafenib, offering new therapeutic strategies. Treatment of various human HCC cell lines with sorafenib/mTKIs downregulated DDX5 in vitro and in preclinical HCC models. Conversely, DDX5 overexpression reduced the viability of sorafenib-treated cells via ferroptosis, suggesting a role for DDX5 in sorafenib sensitivity. RNAseq of wild-type *vs*. DDX5-knockdown cells treated with or without sorafenib identified a set of common genes repressed by DDX5 and upregulated by sorafenib. This set significantly overlaps with Wnt signaling genes, including *Disheveled-1 (DVL1)*, an indispensable Wnt activator and prognostic indicator of poor survival for sorafenib-treated patients. DDX5-knockout (DDX5^KO^) HCC cells exhibited *DVL1* induction, Wnt/β-catenin pathway activation, and ferroptosis upon inhibition of canonical Wnt signaling. Consistently, xenograft HCC tumors exhibited reduced growth by inhibition of Wnt/β-catenin signaling via induction of ferroptosis. Significantly, overexpression of DDX5 in HCC xenografts repressed *DVL1* expression and increased ferroptosis, resulting in reduced tumor growth by sorafenib. We conclude that DDX5 downregulation by sorafenib mediates adaptive resistance by activating Wnt/β-catenin signaling, leading to ferroptosis escape. Conversely, overexpression of DDX5 in vivo enhances the anti-tumor efficacy of sorafenib by suppressing Wnt/β-catenin activation and induction of ferroptosis. Thus, DDX5 overexpression in combination with mTKIs is a promising therapeutic strategy for HCC.

## Introduction

Hepatocellular carcinoma (HCC) is a primary cancer with increasing global incidence [1]. Curative treatments for early-stage HCC of all etiologies include surgical resection, liver transplantation, and percutaneous ablation. In advanced HCCs, multi-tyrosine kinase inhibitors (mTKIs), sorafenib [2] or lenvatinib [3], followed by regorafenib [4], cabozantinib [5], and the anti-angiogenic monoclonal antibody ramucirumab [6] impact on patient survival, but the overall benefit is limited by primary or secondary resistance. The success of combination therapy targeting both VEGF (bevacizumab) and PD-L1 (atezolizumab) [7] and its tolerability [8] have led to its adoption as a first-line treatment. However, mTKIs are still widely used in patients with advanced HCC experiencing contra-indications to immunotherapy [9]. Elucidating the mechanism of mTKI sensitivity will guide the development of new therapeutic strategies to improve mTKI anti-tumor efficacy.

Various mechanisms of sorafenib resistance have been identified, including crosstalk between PI3K/AKT and JAK/STAT pathways, activation of hypoxia-inducible pathways, and epithelial-mesenchymal transitions [10, 11]. Sorafenib response is associated with evasion from ferroptosis [12, 13]. Ferroptosis, a non-apoptotic regulated cell death mechanism, involves membrane lipid peroxidation by ferrous iron (Fe2+) under conditions of increased reactive oxygen species (ROS) [14]. Oxidation of polyunsaturated fatty acid-containing phospholipids, an iron-dependent process, results in the formation of lipid peroxidation by-products such as malondialdehyde (MDA) and 4-hydroxynonenal (4-HNE) [15] which are hallmarks of ferroptosis [16]. In cancer cells, glutathione peroxidase 4 (GPX4) converts lipid hydroperoxides to lipid alcohols, thereby reducing lipid peroxidation, membrane oxidative damage, and ferroptosis [17].

Dysregulation of RNA binding proteins (RBPs) has been identified in several types of cancers [18]. The RBP DDX5 is a DEAD-box RNA helicase [19]. DEAD box helicases unwind RNA duplexes, displace proteins from RNA, remodel RNA-protein complexes, and participate in all aspects of RNA biology [19, 20]. DDX5 is a transcriptional regulator with critical roles in cell growth and differentiation [21], and exhibits diverse functions [19]. In transformed hepatocytes, DDX5 regulates the function of the Polycomb repressive complex 2 (PRC2) [22], and also regulates STAT1 translation by resolving a G-quadruplex located in the 5’UTR of STAT1 mRNA [23]. HCC cell lines with stable DDX5 knockdown (DDX5^KD^) exhibit reduced sensitivity to sorafenib [24] by an unknown mechanism. Herein, we present evidence that DDX5 deficiency orchestrates the activation of Wnt/β-catenin signaling in sorafenib-treated cells, thereby mediating escape from ferroptosis, a mechanism linked to drug resistance in cancer [25, 26].

Analyses of normal human liver and HCCs showed that reduced DDX5 expression was associated with increased tumor grade and worse overall survival of patients treated with sorafenib. Intriguingly, sorafenib reduced the expression of DDX5 in human HCC cell lines and preclinical HCC models, while overexpression of DDX5 in sorafenib-treated cells reduced viability by induction of ferroptosis. Comparison of the transcriptome of wild-type (WT) vs. DDX5-knockdown (DDX5^KD^) HCC cells, treated with or without sorafenib, identified more than 300 genes mutually repressed by DDX5 and induced by sorafenib. KEGG pathway analyses of these common upregulated genes identified the Wnt pathway among the top-ten predicted pathways. Wnt signaling is associated with cancer stem cell renewal [27–29], contributing to poor prognosis and immunosuppression [30–32]. Moreover, Wnt signaling is involved in all aspects of liver development e.g., zonation, regeneration, and homeostasis [33], and is relevant to HCC pathogenesis and drug resistance [34, 35]. Recent studies have linked Wnt/β-catenin activation to ferroptosis escape and chemotherapy (cisplatin) resistance in gastric cancers [36]. Accordingly, we focused on the role of DDX5 and Wnt/ β-catenin activation. We show DDX5 downregulation or DDX5-knockout (DDX5^KO^) increased expression of *DVL1*, indispensable for Wnt activation, and ferroptosis escape in response to sorafenib. *DVL1* overexpression is associated with worse overall survival of patients treated with sorafenib, linking our observations to clinical data. Notably, inhibition of Wnt/ β-catenin signaling or overexpression of DDX5 in a preclinical HCC model improved the anti-tumor efficacy of sorafenib, reducing tumor growth. These results identify DDX5 overexpression as a novel therapy to enhance the anti-tumor efficacy of mTKIs in the treatment of advanced HCC.

## Materials and methods

### Cell culture

Human HCC cell lines utilized include: WT HepAD38 [37], DDX5-knockdown (DDX5^KD^)-HepAD38 [24], Dox-inducible HepaRG-FLAG-DDX5 [23], Dox-inducible Huh7-FLAG-DDX5, HepAD38-FLAG-DDX5 grown as described [23], Dox-inducible Huh7-DVL1 and HepAD38-DVL1 cell lines; SNU387, SNU423, Hep3B, Huh7, and HepaRG grown according to ATCC recommendations. Cell lines were routinely tested for mycoplasma. HepAD38 cell lines were authenticated by short tandem repeat (STR) analysis.

### CRISPR/Cas9 gene editing

Huh7 cells were used to introduce indels targeting exon 2 of the DDX5 gene, using CRISPR/Cas9 system. Ribonucleoprotein of Cas9-2NLS (10 µmol, Synthego) and guide RNA (100 pmol, Synthego) were electroporated into 1.2 × 10^5^ cells, using Neon Transfection System at 1200 V, for 20 ms and four pulses (ThermoFisher Scientific), according to manufacturer’s instructions. The incorporation of indels was determined using genomic DNA isolated 48 h after electroporation and rapid polyacrylamide gel electrophoresis-based (PAGE) [38]. Primers used for the rapid PAGE genotyping method: fwd 5’-AACCTGGGTATAGCCATTTGAA-3’, rev 5’-CCTGATGAAGCCACATGAATTTAC-3’. Validated pools of cells were subjected to clonal selection. The genomic DNA of individual clones was analyzed by polymerase chain reaction (PCR) and DNA sequencing of purified PCR products.

### Transfection assays

HCC cell lines (5 × 10^4^ cells) transfected with 100 ng of Wnt-Reporter TOPFlash (TCL/LEF-Firefly Luciferase) vector and Renilla luciferase (100 ng). Indicated siRNAs (50 pM) transfected using RNAiMax (Life Technologies). Luciferase activity was measured 48 h after transfection using the Dual Luciferase Assay System, according to the manufacturer’s instructions (Promega), and normalized to Renilla luciferase. Plasmids and siRNAs are listed in Supplementary Table S1.

### C11-BODIPY ^581/591^ assay

Cells were seeded into a 29 mm glass bottom dish with 14 mm micro-well #1.5 cover glass and treated with DMSO (vehicle), sorafenib (15 μM), or siRNAs transfected for 24 h, as indicated. Cells were labeled with 5.0 μM C11-BODIPY ^581/591^ (Life Technologies) at 37 °C for 10 min and visualized by fluorescence microscopy at 510 nm and 590 nm.

### Lipid peroxidation assays

*Malondialdehyde (MDA)* (ab233471) and *4-hydroxynonenal (4-HNE)* (ab238538) assays were carried out as described by manufacturer (Abcam).

### Cell viability assays

HCC cells (1 × 10^4^) seeded in 96-well plates treated with DMSO, sorafenib (7.5–10 μM), ferrostatin (10 µM), Z-VAD-FMK (10 µM), necrosulfonamide (4.0 µM), or transfected with siRNAs (50 pM) for 24 h. Growth inhibition was measured at 490 nm using the CellTiter 96 AQ_ueous_ One Solution Cell Proliferation assay, a 3-(4,5-dimethylthiazol-2-yl)-5-(3-carboxymethoxyphenyl)-2-(4-sulfophenyl)-2H-tetrazolium (MTS)-based assay (Promega). Viability (100%) refers to A_490_ value of DMSO-treated cells. Background absorbance was measured in wells containing medium and MTS.

### Huh7 xenografts

Tumor xenografts were established by subcutaneous injection of 5 × 10^6^ Huh7 cells per NRG mouse. When tumors reached a mean volume of ∼70–100 mm^3^, mice were randomized to control and treated groups, and received vehicle (5% DMSO + 45% PEG400) or sorafenib orally at 40 mg/kg daily for the first 7 days, followed by 80 mg/kg daily for remaining 2 weeks. Huh7 DDX5 overexpressing tumor-bearing mice were generated using Dox-inducible Huh7-FLAG-DDX5 cells. Doxycycline-containing H_2_O (1.0 µg/ml) was fed to half the mice, 48 h prior to daily administration of sorafenib (80 mg/kg, 5 days/week), when tumor volume reached 50–70 mm^3^.

### HBx/c-Myc mice

Bi-transgenic HBx/c-Myc mice were maintained at the Cancer Research Center of Lyon (CRCL), France. Twenty-week-old mice (4 males and 12 females) were injected with Exitron nano 6000 contrast agent (Miltenyi Biotech), and liver tumor growth was monitored by micro-computerized tomography (µCT) once a week. Animals with a tumor diameter of 2 mm were randomized into sorafenib-treated or vehicle groups. Sorafenib or vehicle administered by oral gavage five times per week. µCT monitoring continued until the animals died. The liver nodules measured included those that appeared after the onset of treatment. Animals were sacrificed at 6 weeks of treatment or when the tumor diameter was more than 12 mm (ethical euthanasia). Peritumor tissues and tumors are excised and frozen at −80 °C or fixed in formalin. Sections were stained with DDX5 antibody (Supplementary Table S2) and counterstained with hematoxylin and eosin using the CRCL pathology platform.

### Nanosac preparation

Nanosacs carrying siCtrl or siβ-catenin were prepared as previously described [39]. Nanosac-encapsulated siRNAs were administered every 48 h intra-tumorally, delivering 3.0 µg siRNA per injection. Detailed protocol for Nanosac preparation is included in Supplementary Materials.

*Immunoblotting* is performed as described in Supplementary Materials. Antibodies used are listed in Supplementary Table S2.

*Immunohistochemistry* assays are performed as described [23].

### RNA preparation and qRT-PCR

Methods included in Supplementary Materials; primer sequences listed in Supplementary Table S3, and reagents, chemical inhibitors, and kits in Supplementary Table S4.

### RNA-seq analysis

Detailed methods of transcriptomic analyses of WT HepAD38 [37] and DDX5^KD^ cells [24] treated with sorafenib are included in Supplementary Materials. Gene set enrichment analysis (GSEA) was performed using GSEA software [40].

### Statistical analysis

Statistical analysis was performed using an unpaired *t*-test in GraphPad Prism (version 6.0; GraphPad Software, San Diego, CA, USA). Differences were considered statistically significant at *p* < 0.05.

## Results

### DDX5 deficiency associated with increased HCC grade and reduced patient survival in response to sorafenib

**Fig. 1:**
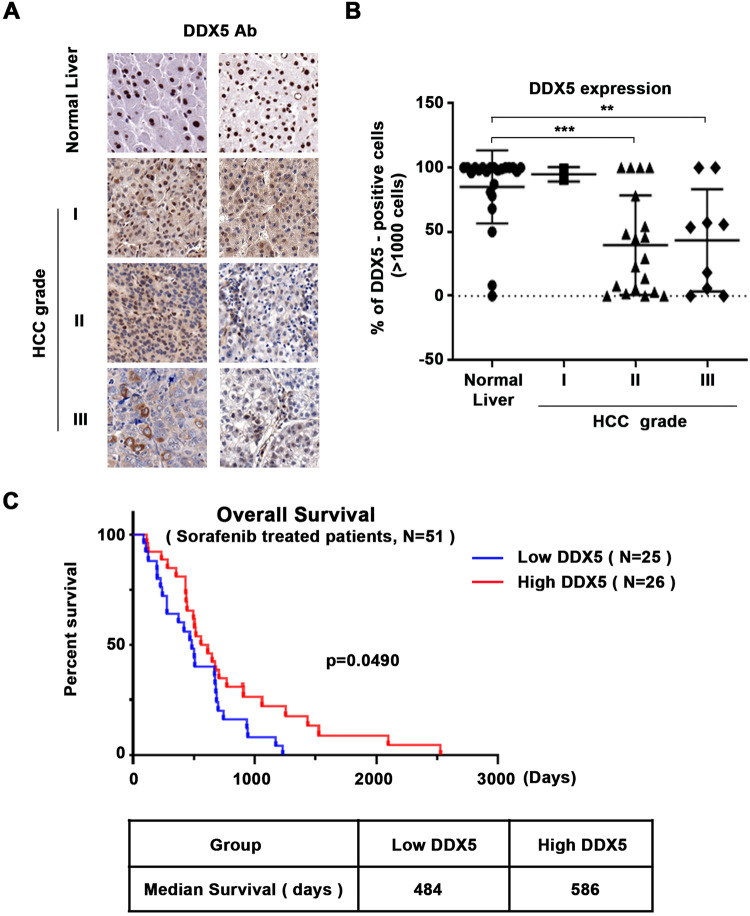
Reduced DDX5 protein levels in human HCCs associated with increased tumor grade and poor patient survival following sorafenib treatment. **A** Immunohistochemistry (IHC) with DDX5 antibody of tissue microarrays (TMA) comprised of human normal liver samples and HCCs of grades I-III. Representative images at 20× magnification. IHC images of TMAs (24 normal samples and 30 HCCs) are shown in Supplementary Figs. S1A and S2–S3. **B** Quantification of DDX5-positive cells from TMAs of normal liver and HCCs (>1000 cells were quantified per tumor). **p < 0.01 and ***p < 0.001 by unpaired *t*-test. **C** Overall survival of patients treated with sorafenib; the red line indicates patients with high DDX5, and the blue line indicates patients with low DDX5, quantified from IHC images using a NanoZoomer 2.0 RS Pathology slide scanner (C10730-13, Hamamatsu) and NDP.view2 Image viewing software (U12388-01, HAMAMATSU), as described in Supplementary Materials and Methods.

Our earlier studies suggested a role for DDX5 in poor prognosis HCC [22], and that DDX5 knockdown enables HCC cells to form hepatospheres, exhibiting growth insensitive to sorafenib [24] by an unknown mechanism. Herein, we determined by immunohistochemistry (IHC) the expression of DDX5 in human HCCs, using a commercially available tissue microarray (US Biolab Corporation, Inc.). In agreement with earlier results [22], HCC tumors of grade II and III, exhibited a statistically significant reduction in the number of hepatocytes with DDX5-positive immunostaining compared to normal liver tissue (Fig. 1A, B and Supplementary Figs. S1A–S3). Similarly, we analyzed DDX5 by IHC of 51 HCCs from patients treated with sorafenib. Reduced immunostaining for DDX5 is associated with reduced patient survival following treatment with sorafenib (Fig. 1C and Supplementary Fig. S1B), suggesting a role for DDX5 in the sorafenib response.

### Sorafenib downregulates DDX5 in transformed hepatocytes in vitro and in vivo

**Fig. 2:**
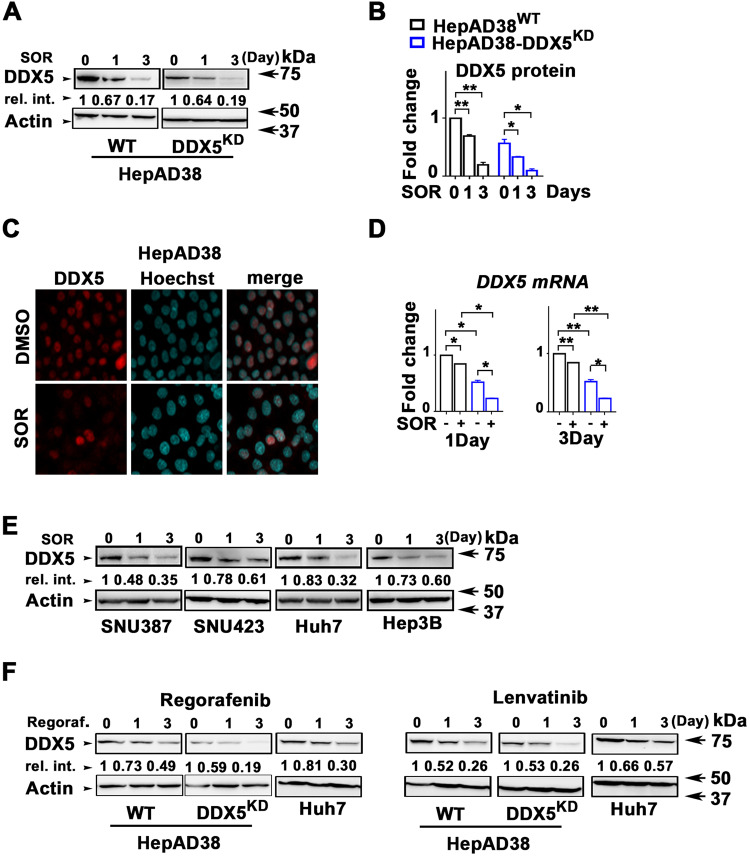
Sorafenib (SOR) downregulates DDX5 in vitro. **A** Immunoblots of DDX5 using lysates from WT and DDX5^KD^ HepAD38 cells was treated with sorafenib (SOR) (10 µM for 1 day and 7.5 µM for 3 days). Actin used as loading control **B** Quantification of DDX5 levels from immunoblots by ImageJ software. Error bars represent the standard deviation (SD) from three independent experiments (*n* = 3). *p < 0.05, **p < 0.01 by unpaired *t-*test. **C** Immunofluorescence microscopy of DDX5 in HepAD38 cells treated with SOR (10 µM) for 1 day. **D** RT-PCR quantification of *DDX5* mRNA using RNA from WT and DDX5^KD^ HepAD38 cells treated with sorafenib (SOR) (10 µM for 1 day and 7.5 µM for 3 days). Data expressed as mean ± standard error of the mean (SEM), *n* = 3. *p < 0.05, **p < 0.01 by unpaired *t-*test. **E**, **F** Immunoblots of DDX5 using lysates from indicated cell lines treated with **E** SOR (SNU387 and SNU423: 15 µM for 1 day and 10 µM for 3 days; Huh7 and Hep3B: 10 µM for 1 day and 5 µM for 3 days), and **F** regorafenib (10 µM), lenvatinib (50 µM), as indicated. Shown, are representative immunoblots from *n* = 3. Actin is used as a loading control.

**Fig. 3:**
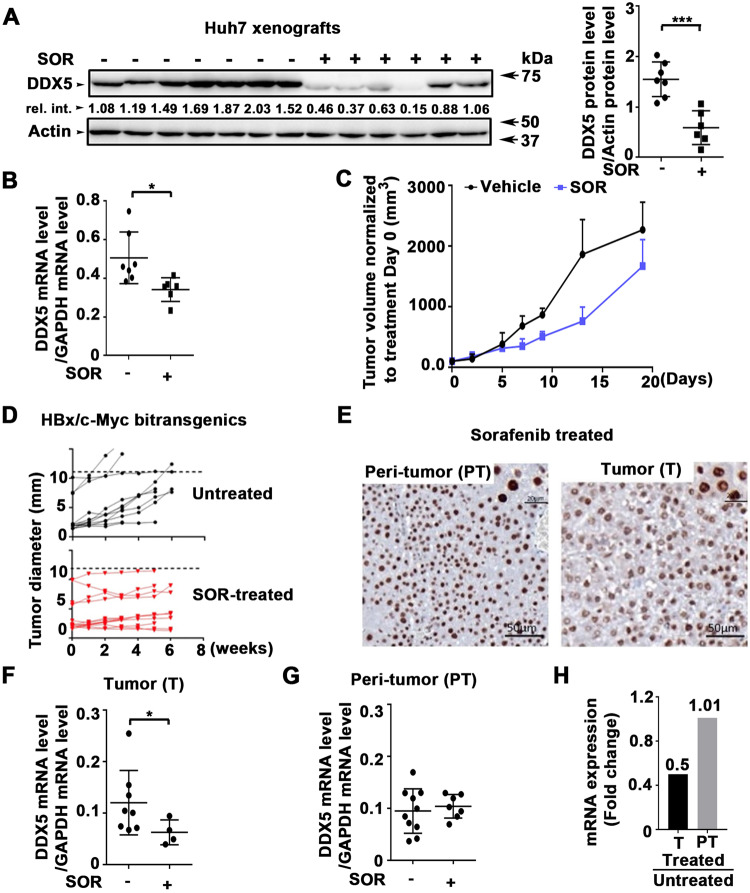
Sorafenib downregulates DDX5 in preclinical HCC models. **A**–**C** Huh7 xenografts. NRG mice bearing Huh7 tumors were treated daily with 40 mg/kg sorafenib (SOR) for 1 week followed by 80 mg/kg SOR for 2 weeks (+) or DMSO (−) for 20 days. **A** DDX5 immunoblots from Huh7 tumors ± SOR, as indicated. Quantification of DDX5 protein levels from immunoblots by ImageJ software. Error bars represent SD from eight tumors. ***p < 0.001 by unpaired *t-*test. Actin is used as a loading control. **B** Quantification of *DDX5* mRNA by qRT-PCR in tumors +/- SOR. Data are expressed as mean ± SEM from eight tumors. *p < 0.05 by unpaired *t-*test. **C** Tumor volume ± SOR normalized to day 0 of treatment. **D**–**H** HBx/c-myc mice. **D** Tumor growth was monitored by µCT scanner from each group, untreated (DMSO) and SOR-treated (60 mg/kg), as indicated. **E** Immunohistochemistry of formalin-fixed paraffin-embedded *(*FFPE) tumor and peri-tumor stained with DDX5 antibody, and counterstained with hematoxylin. **F**–**H** RT-qPCR detection of mRNA levels of *DDX5* in (**F**, **G**) SOR-treated vs. untreated (DMSO) **F** tumors and **G** peri-tumoral tissue from HBx/c-Myc mice. *p < 0.05. **H** *DDX5* mRNA level expressed as fold change between SOR-treated and untreated tumors and peri-tumoral tissue.

Intriguingly, we found that sorafenib treatment, 1–3 days, of HepAD38 cells and various human liver cancer cell lines resulted in progressive downregulation of DDX5 (Fig. 2A–E). Similarly, mTKIs regorafenib and lenvatinib also progressively downregulated DDX5 (Fig. 2F). To confirm these in vitro observations, we used two preclinical models of HCC (Fig. 3). Huh7 xenografts in immunocompromised NRG mice and the murine HCC model of HBx/c-Myc bitransgenics [41]. HBx/c-Myc bitransgenics develop liver tumors at 5–7 months without treatment with hepatocarcinogens [41], resembling human HCCs with a progenitor phenotype [42].

Mice bearing Huh7 tumors were treated with vehicle or sorafenib daily for 20 days. DDX5 expression in untreated and treated xenografts was quantified by immunoblotting (Fig. 3A) and mRNA by qRT-PCR (Fig. 3B). Sorafenib significantly reduced DDX5 expression in vivo, but did not significantly affect tumor volume (Fig. 3C). Similarly, HBx/c-Myc mice (20 weeks old) received sorafenib 5 days/week for 6 weeks. Liver tumor growth in the HBx/c-Myc mouse model as a function of sorafenib showed, as in Huh7 xenografts (Fig. 3C) and similar to what is observed in HCC patients, reduction of tumor growth rate, without significant tumor regression (Fig. 3D). IHC of DDX5 showed a higher number of nuclei with “diffuse”/less intense DDX5 staining in sorafenib-treated tumors than in peri-tumor (Fig. 3E), consistent with reduced DDX5 mRNA levels in sorafenib-treated tumors (Fig. 3F, H), but not in the peri-tumoral tissue (Fig. 3G, H).

### Sorafenib-induced ferroptosis mediated by DDX5 in HCC cells

**Fig. 4:**
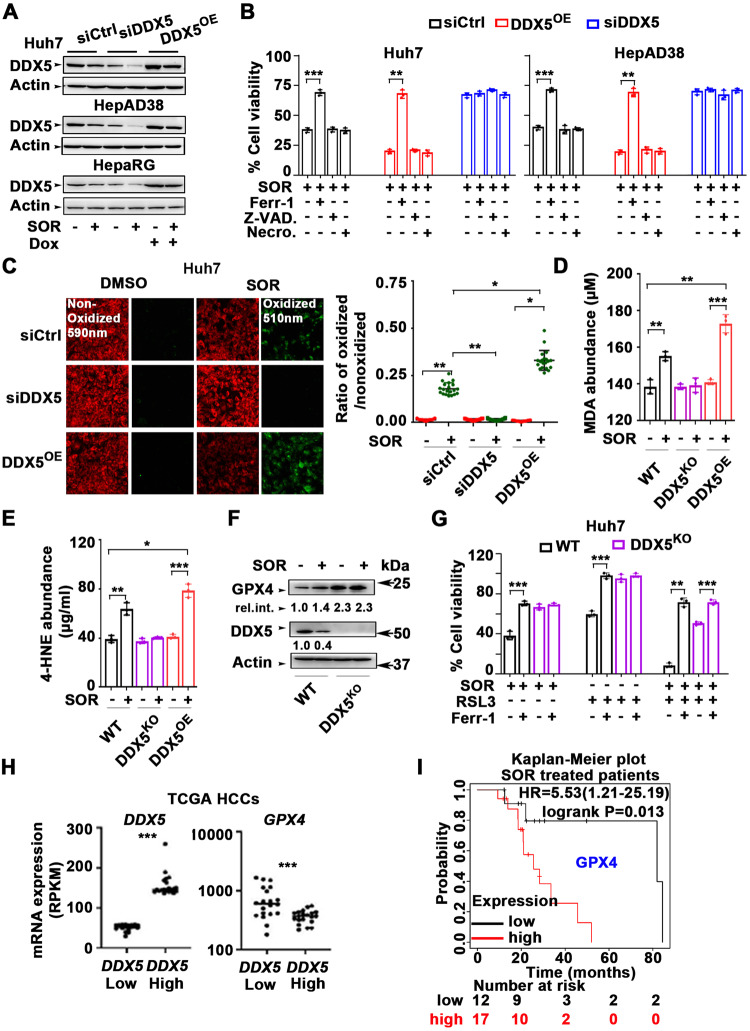
DDX5 regulates ferroptosis in sorafenib-treated cells. **A** Immunoblots of DDX5 in indicated cell lines. Huh7, HepAD38, and HepaRG cell lines transfected with siCtrl or siDDX5 for 24 h, followed by addition for 24 h of SOR (10 µM for Huh7 and 15 µM for HepAD38 and HepaRG cell lines). For DDX5^OE^, indicated Dox-inducible-DDX5 cell lines were grown with Dox (1.0 µg/ml) for 48 h, and SOR for the last 24 h. A representative immunoblot is shown, *n* = 3 Quantification of immunoblots by ImageJ software shown in supplementary Fig. S4A. **B** Cell viability of Huh7 and HepAD38 cells under conditions of siCtrl, siDDX5 or DDX5^OE^ as described in (**A**), treated with SOR, ±10 µM ferrostatin-1 (Ferr-1), ±Z-VAD-FMK (10 µM) or ±necrosulfonamide (4.0 µM) for 24 h. Data expressed as mean ± SEM, *n* = 3. *p < 0.05, **p < 0.01 by unpaired *t-*test. **C** Fluorescence microscopy of C11-BODIPY using Huh7 cells, under conditions of siCtrl, siDDX5, or DDX5^OE^ as described in (**A**), treated ±SOR (10 µM) for 24 h. (Right panel) Quantification by ImageJ software of the ratio of oxidized (510 nm)/non-oxidized (590 nm) C11-BODIPY. Data expressed as mean ± SEM from >1000 cells per condition. *p < 0.05, ** *p* < 0.01 by unpaired *t-*test. **D** MDA abundance (µM) quantified using lysates from Huh7 wild type (WT), DDX5^KO^, and DDX5^OE^ cells treated as described in (**A**), without (−) or with (+) SOR for 24 h. Data are expressed as SD, *n* = 3. **E** 4-HNE abundance (µg/ml) quantified using lysates from WT, DDX5^KO^, and DDX5^OE^ Huh7 cells treated as described in (**A**), without (−) or with (+) SOR for 24 h. Data are expressed as SD, *n* = 3. **F** Immunoblots of GPX4 and DDX5, as indicated, using lysates from WT and DDX5^KO^ Huh7 cells grown without (−) or with (+) SOR for 24 h. Relative intensity is quantified vs. actin. A representative experiment is shown from *n* = 3. **G** Cell viability of WT and DDX5^KO^ Huh7 cells treated with SOR, RSL3 (0.5 µM) or Ferr-1 (10 µM), as indicated, for 24 h. Data expressed as mean ± SEM, *n* = 3. *p < 0.05, **p < 0.01 by unpaired *t-*test. **H** Dot plots showing expression of *DDX5* and *GPX4* mRNAs in HCCs from TCGA with lowest vs. highest *DDX5* expression. Twenty HCCs were analyzed per group. Median highlighted, ***p < 0.001. **I** Kaplan–Meier survival plots for *GPX4* expression of SOR treated patients with HCC. HCC samples (*n* = 29) are from TCGA.

Sorafenib response in in vitro and preclinical HCC models is improved by pharmacological induction of ferroptosis [12]. Since DDX5^KD^ cells are insensitive to sorafenib [24], we hypothesized that DDX5 plays a role in ferroptosis. To test this possibility, we generated doxycycline (Dox)-inducible DDX5 expressing cell lines (DDX5^OE^) in Huh7, HepAD38, and HepaRG [23] cells, representing distinct transformation and differentiation states. First, DDX5 protein levels were quantified with or without sorafenib in Huh7, HepAD38, and HepaRG cells transfected with control siRNA (siCtrl) or siRNA targeting DDX5 (siDDX5), as well as in DDX5 overexpressing (DDX5^OE^) cells following Dox addition (Fig. 4A and Supplementary Fig. S4A). Employing these DDX5 expression conditions, we quantified cell viability in response to sorafenib. siDDX5 significantly enhanced cell viability (Supplementary Fig. S4B, C), whereas DDX5^OE^ significantly sensitized cells to sorafenib, reducing cell viability in the three HCC cell lines tested (Supplementary Fig. S4D).

Next, we determined the type of regulated cell death rescued by siDDX5 in sorafenib-treated cells. Ferroptosis inhibitor ferrostatin-1 (Ferr-1) [14] rescued the viability of Huh7 and HepAD38 cells as well as of the corresponding DDX5^OE^ cells, whereas apoptosis-specific inhibitor Z-VAD-FMK and necroptosis-specific inhibitor necrosulfonamide did not (Fig. 4B). In addition, siDDX5 decreased sorafenib sensitivity, independently of Ferr-1, Z-VAD-FMK, or necrosulfonamide (Fig. 4B). Using C-11 BODIPY, a lipid-soluble fluorescent indicator of lipid oxidation, and established surrogate for quantifying ferroptosis [43], we observed siDDX5 reduced, and DDX5^OE^ increased lipid oxidation (Fig. 4C and Supplementary Fig. S4E). To corroborate these findings by an alternative method, we generated a DDX5-knockout (DDX5^KO^) Huh7 cell line by CRISPR/Cas9 gene editing (Supplementary Fig. S5A, B). We quantified the level of malondialdehyde (MDA), a marker of oxidation of polyunsaturated fatty acid-containing phospholipids and a hallmark of ferroptosis, in WT Huh7 cells, Huh7-DDX5^KO^ and Huh7-DDX5^OE^ cells, with or without sorafenib treatment [16], (Fig. 4D and Supplementary Fig. S5C). Sorafenib increased MDA levels in WT Huh7 and Huh7-DDX5^OE^ but not in Huh7-DDX5^KO^ cells, demonstrating that DDX5 loss abolished lipid peroxidation and ferroptosis. Similar results were obtained by quantifying the level of 4-hydroxynonenal (4-HNE) (Fig. 4E and Supplementary Fig. S5D), also a by-product of lipid peroxidation and a stable ferroptosis marker [16].

Since GPX4 reduces lipid peroxidation and ferroptosis [44], we assessed GPX4 levels in WT Huh7 and Huh7-DDX5^KO^ cells by immunoblots. Sorafenib increased GPX4 levels in WT Huh7 cells, and notably, Huh7-DDX5^KO^ exhibited enhanced GPX4 levels independent of sorafenib addition (Fig. 4F). To assess the functional significance of GPX4 induction in Huh7-DDX5^KO^ cells, we quantified survival of cells treated with sorafenib in combination with the class-II ferroptosis inhibitor RSL3, which binds and inactivates GPX4 [17]. RSL3 reduced cell survival of sorafenib-treated WT Huh7 and Huh7-DDX5^KO^ cells, which was restored by ferrostatin addition (Fig. 4G). Similar results were observed in WT and DDX5^KD^ HepAD38 cells (Supplementary Fig. S5E-F). Moreover, Huh7 xenograft tumors treated with sorafenib exhibited enhanced *GPX4* mRNA (Supplementary Fig. S5G). Additionally, siDDX5 further enhanced *GPX4* mRNA levels upon sorafenib addition (Supplementary Fig. S5H, I), suggesting that DDX5 exerts a role in *GPX4* transcription.

To determine the clinical relevance of these observations, we measured *DDX5* and *GPX4* mRNA levels in human HCC samples from TCGA. We found that HCCs with low *DDX5* mRNA exhibited higher *GPX4* mRNA compared to HCCs with high *DDX5* expression (Fig. 4H), and importantly, elevated *GPX4* mRNA levels were associated with poor survival outcomes in sorafenib-treated HCC patients (Fig. 4I).

Together, these results link DDX5 to ferroptosis, in response to sorafenib. Since DDX5 is an RNA helicase, we employed Dox-inducible FLAG-DDX5-HepaRG cell lines, encoding WT or ATPase-inactive K144N DDX5 mutant [22, 23] to determine whether the enzymatic activity of DDX5 is required for ferroptosis. We found WT DDX5 induced ferroptosis by sorafenib, while the inactive K144N DDX5 did not affect lipid peroxidation, i.e., cells escaped ferroptosis (Supplementary Fig. S5J). These results demonstrate the enzymatic RNA helicase activity of DDX5 is required for ferroptosis, by a mechanism that remains to be determined.

### Sorafenib-induced DDX5 downregulation increased the expression of Wnt/β-catenin signaling genes

**Fig. 5:**
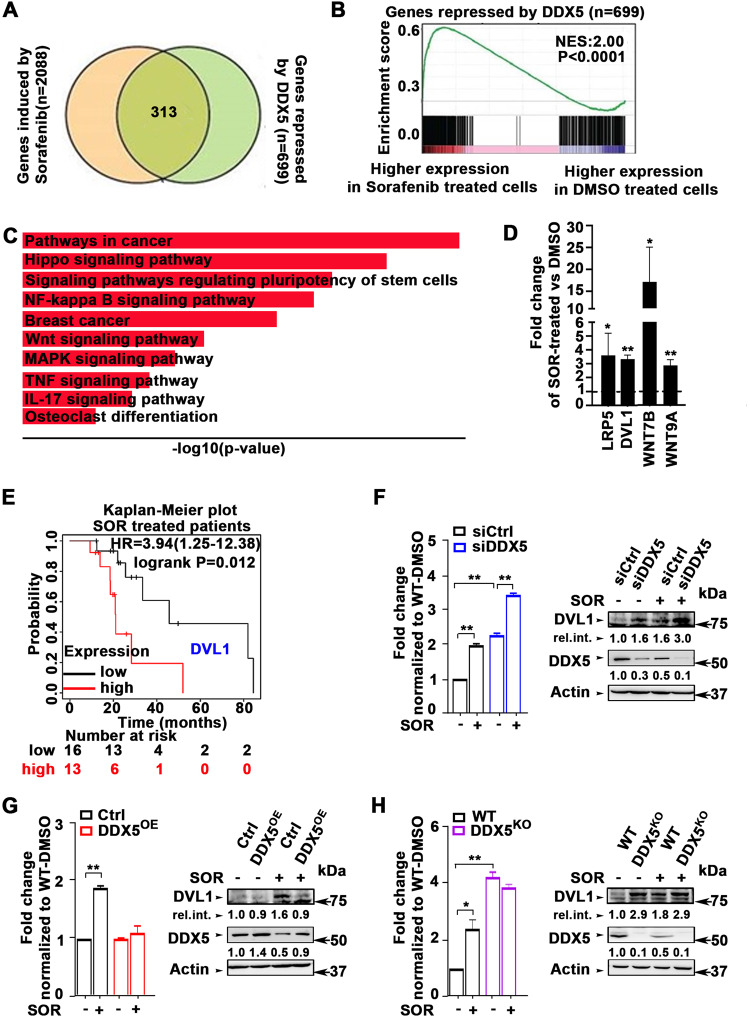
Sorafenib-induced and DDX5-repressed genes enriched in Wnt/β-catenin signaling. **A** Venn diagram of common genes between SOR-induced and DDX5-repressed genes. **B** GSEA plot showing enrichment of genes more highly expressed in SOR vs. DMSO treated WT HepAD38 cells, and repressed by DDX5. **C** Top 10-most enriched KEGG pathways associated with genes induced by SOR and repressed by DDX5. **D** qRT PCR of indicated Wnt/β-catenin signaling genes using RNA from HepAD38 cells treated with sorafenib (7.5 µM) for 3 days. Data are mean ± SEM, *n* = 3. *p < 0.05, **p < 0.01 by unpaired *t-*test. **E** Kaplan–Meier survival plots for *DVL1* expression of SOR treated patients with HCC. Samples are from TCGA. **F**–**H** qRT-PCR of *DVL1* mRNA and immunoblots of DVL1 protein, using total RNA or lysates, respectively, isolated from: **F** WT Huh7 cells transfected with siCtlr or siDDX5, **G** WT and DDX5^OE^ Huh7 cells, grown as described in Fig. 4A, and **H** WT and DDX5^KO^ Huh7 cells, ±SOR (10 µM) for 24 h, as indicated. qRT-PCR data are expressed as mean ± SEM from *n* = 3. *p < 0.05, **p < 0.01 by unpaired *t-*test. A representative DVL1 immunoblot is shown *n* = 3. Actin is used as a loading control.

To identify cellular pathways deregulated by sorafenib-induced downregulation of DDX5, we compared transcriptomes of WT and DDX5^KD^ HepAD38 cells treated or not treated with sorafenib. We identified 2,088 genes significantly induced by sorafenib, and 699 genes significantly repressed by DDX5, of which 313 genes were shared, that is, those induced by sorafenib and repressed by DDX5 (Fig. 5A). Gene set enrichment analysis (GSEA) revealed that genes repressed by DDX5 were enriched in genes highly expressed in sorafenib-vs. DMSO-treated WT HepAD38 cells (Fig. 5B), suggesting a significant number of sorafenib-regulated genes are also regulated by DDX5. KEGG pathway analysis of the common 313 genes identified among the top ten associated pathways the Wnt signaling pathway (Fig. 5C). To confirm sorafenib increased expression of Wnt signaling genes, we analyzed RNA isolated from WT HepAD38 cells treated with sorafenib for 3 days, using a PCR array comprising >90 Wnt signaling genes. Sorafenib increased the expression of many Wnt signaling genes (Supplementary Fig. S6A), including *LRP5, DVL1, Wnt7B*, and *Wnt9A* (Fig. 5D), indispensable for Wnt pathway activation.

Notably, using TCGA HCCs, we found that high *DVL1* expression was associated with poor survival of sorafenib-treated patients (Fig. 5E). Based on this observation, we investigated the regulation of Wnt signaling genes by DDX5 by focusing on *DVL1*. We observed siDDX5 increased *DVL1* mRNA and protein (Fig. 5F), while DDX5^OE^ fully repressed DVL1 induction (Fig. 5G and Supplementary Fig. S6C). Sorafenib also increased DVL1 expression, and this increase was higher in siDDX5 cells (Fig. 5F and Supplementary Fig. S6B). To demonstrate whether sorafenib-mediated induction of *DVL1* was solely via DDX5 downregulation, we determined DVL1 expression in the Huh7-DDX5^KO^ cell line. In DDX5^KO^ cells *DVL1* mRNA and DVL1 protein levels increased independently of sorafenib (Fig. 5H), thereby demonstrating that DDX5 is an upstream negative regulator of *DVL1* transcription.

### Activation of Wnt/β-catenin is required for DDX5-mediated ferroptosis escape of sorafenib-treated cells

**Fig. 6:**
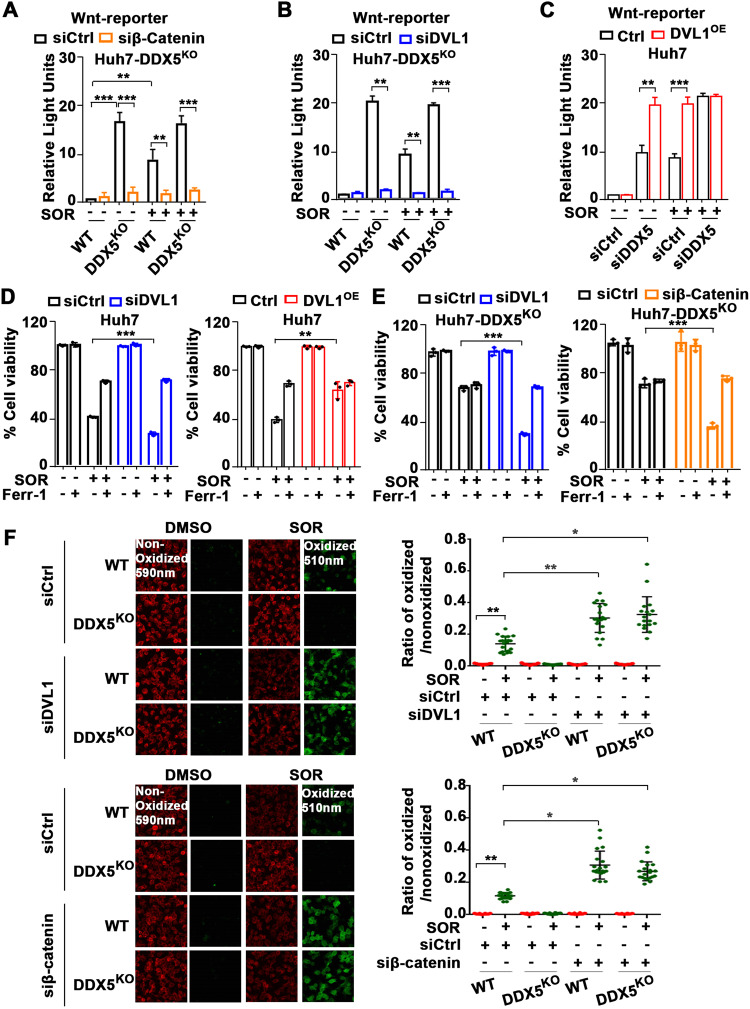
Activation of Wnt/β-catenin signaling mediates ferroptosis escape of DDX5 deficient cells by sorafenib. Wnt-reporter (TOPFlash) and Renilla-luciferase plasmids (100 ng each per 12-well plate) co-transfected in WT and DDX5^KO^ (**A**, **B**) and DDX5^OE^ (**C**) Huh7 cells with siRNAs (50 pM each) siCtrl, siβ-catenin, siDVL1 and siDDX5, as indicated, treated ±SOR (10 µM) for 24 h. Data expressed as mean ± SEM, *n* = 3. **p < 0.01 ***p < 0.001 by unpaired *t-*test. Cell viability assays of Huh7 and DVL1^OE^ (**D**), and DDX5^KO^ (**E**) cells transfected with indicated siRNAs, treated with SOR (10 µM), ±Ferr-1 (10 µM) for 24 h. Data expressed as mean ± SEM, *n* = 3. **p < 0.01, ***p < 0.001 by unpaired *t-*test. **F** Fluorescence microscopy of C11-BODIPY using WT and DDX5^KO^ Huh7 cells transfected with siCtrl, siDVL1 or si-β-catenin treated ±SOR (10 µM) for 24 h. Quantification by ImageJ software of the ratio of oxidized (510 nm)/non-oxidized (590 nm) C11-BODIPY. Data are expressed as mean ± SEM from >1000 cells per condition. *p < 0.05, **p < 0.01 by unpaired *t-*test.

Since DVL1 is a key effector of Wnt activation [45] and dysregulated Wnt/β-catenin signaling is implicated in ferroptosis [36], we examined the activation status of the canonical Wnt pathway using the β-catenin-responsive TOPFlash luciferase reporter, as well as the effect of DVL1 on Wnt-reporter expression. As expected, Wnt signaling was activated by DDX5 downregulation [24] and sorafenib addition, while siβ-catenin significantly reduced Wnt-reporter expression (Fig. 6A and Supplementary Fig. S7A). Notably, DDX5^KO^ cells induced Wnt-reporter activation, independent of sorafenib addition (Fig. 6A, B), and siDVL1 completely abolished Wnt-reporter activation (Fig. 6B and Supplementary Fig. S7B). By contrast, DVL1 overexpression (DVL1^OE^), using Dox-inducible DVL1 overexpressing cell lines (Supplementary Fig. S7C), increased Wnt-reporter expression upon siDDX5 transfection or sorafenib treatment (Fig. 6C and Supplementary Fig. S7D). These results demonstrate that DDX5 is an upstream negative regulator of Wnt/β-catenin pathway activation in hepatocytes, and significantly, activation of Wnt signaling by sorafenib involves DVL1 induction through downregulation of DDX5.

**Fig. 7:**
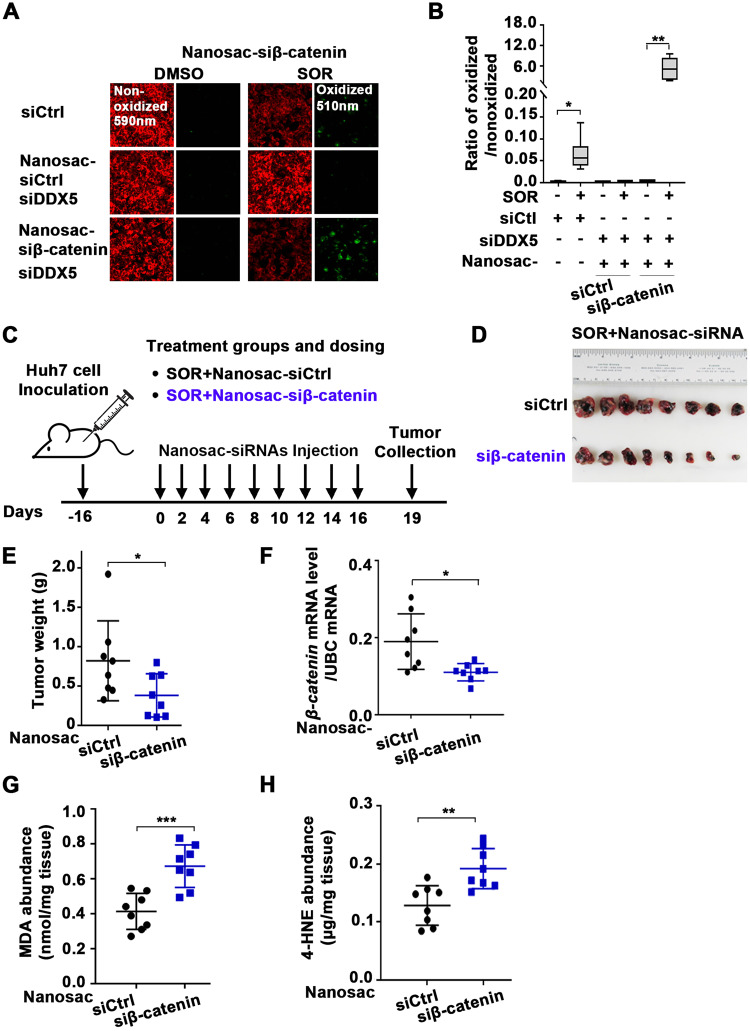
Nanosac-encapsulated siRNA (siβ-catenin) enhances the anti-tumor efficacy of sorafenib. **A** Fluorescence microscopy of C11-BODIPY using Huh7 cells transfected with siDDX5 and incubated with Nanosac-encapsulated siCtrl or siβ-catenin for 24 h, followed by the addition of SOR (10 μM) for 24 h. **B** Quantification by ImageJ software of ratio of oxidized (510 nm)/non-oxidized (590 nm) C11-BODIPY. Data are shown as mean ± SEM from 500 cells. *p < 0.05, **p < 0.01 by unpaired *t-*test. **C** Diagram illustrates treatment groups and timetable of intra-tumoral injection of Nanosac-encapsulated siRNAs. **D** Images of Huh7 xenograft tumors excised on day 19, following three intra-tumoral injections/week of indicated Nanosac-encapsulated siRNAs (3.0 µg siRNA/injection), and daily administration of SOR (80 mg/kg). **E** Tumor weight from indicated treatment groups, from eight tumors. *p < 0.05 by unpaired *t-*test. **F** RT-PCR quantification of *β-catenin* mRNA using total RNA isolated from Huh7 tumors treated with indicated Nanosac-encapsulated siRNAs +SOR. Data are expressed as mean ± SEM from eight tumors.*<p.05 by unpaired *t-*test. Quantification of; **G** MDA (nmole/mg tissue) and **H** 4-HNE (µg**/**mg tissue) using Huh7 xenograft tumors treated as indicated. Data are expressed as mean ± SEM from eight tumors. ***p < 0.001 by unpaired *t-*test.

Since DDX5 downregulation enabled ferroptosis escape of sorafenib-treated cells (Fig. 4B–E and Supplementary Fig. S5C, D) and DDX5^KO^ cells exhibited active Wnt*/*β-catenin signaling (Fig. 7A), we examined whether Wnt*/*β-catenin activation plays a role in ferroptosis escape. Inhibition of Wnt signaling by siDVL1 transfection reduced viability of Huh7 cells treated with sorafenib, while ferrostatin (Ferr-1) reversed this effect (Fig. 6D). By contrast, DVL1 overexpression, using Dox-inducible Huh7-DVL1^OE^ cells, increased cell viability in the presence of sorafenib, independent of ferrostatin (Fig. 6D). We observed similar results with HepAD38 cells (Supplementary Fig. S7E). Importantly, Huh7-DDX5^KO^ cells escaped ferroptosis by sorafenib, determined by cell viability (Fig. 6E, F) and C11-BODIPY assays (Fig. 6G), whereas inhibition of Wnt/β-catenin signaling by transfection of siDVL1 or si-β-catenin reversed this effect (Fig. 6E–G). Similarly, in HepAD38 cells, siDVL1, siβ-catenin, or the canonical Wnt-signaling inhibitors ICG001 and XAV939 [46] suppressed ferroptosis escape and reduced cell viability, respectively (Supplementary Fig. S7E–H). Thus, DDX5 deficiency promotes HCC cell survival to sorafenib through ferroptosis escape by induction of DVL1 and activation of Wnt*/*β-catenin signaling.

### Knockdown of *β-catenin* or DDX5 overexpression increased sorafenib anti-tumor efficacy in xenograft tumors

The mechanistic links between DDX5 and sorafenib sensitivity suggested strategies to enhance the anti-tumor efficacy of sorafenib. First, we investigated the effect of suppressing Wnt/β-catenin activation, utilizing an in vivo siRNA-mediated knockdown of *β-catenin* mRNA. We employed the recently developed Nanosac formulation [39] as a siRNA carrier because Nanosac-encapsulated siRNAs offer effective cytosolic delivery and intracellular release of siRNA without endosomal sequestration [39]. Nanosac-encapsulated siRNA targeting *β-catenin* mRNA was effective in inducing ferroptosis of siDDX5 transfected Huh7 cells treated with sorafenib. C11-BODIPY assays displayed enhanced lipid peroxidation upon incubation with Nanosac-siβ-catenin in comparison to Nanosac-siCtrl, in the presence of sorafenib (Fig. 7A, B). Next, we examined the effect of Nanosac-encapsulated siRNAs in vivo, using Huh7 xenografts co-treated with sorafenib. Intra-tumoral injection of Nanosac-siβ-catenin in combination with sorafenib (Fig. 7C) significantly reduced tumor weight (Fig. 7D, E) and *β-catenin* mRNA levels (Fig. 7F) compared to Nanosac-siCtrl. By contrast, the level of lipid peroxidation by-products MDA and 4-HNE, both markers of ferroptosis [16], were significantly increased (Fig. 7G, H). Thus, siRNA interfering with Wnt/β-catenin activation enhanced the anti-tumor efficacy of sorafenib in vivo.

**Fig. 8:**
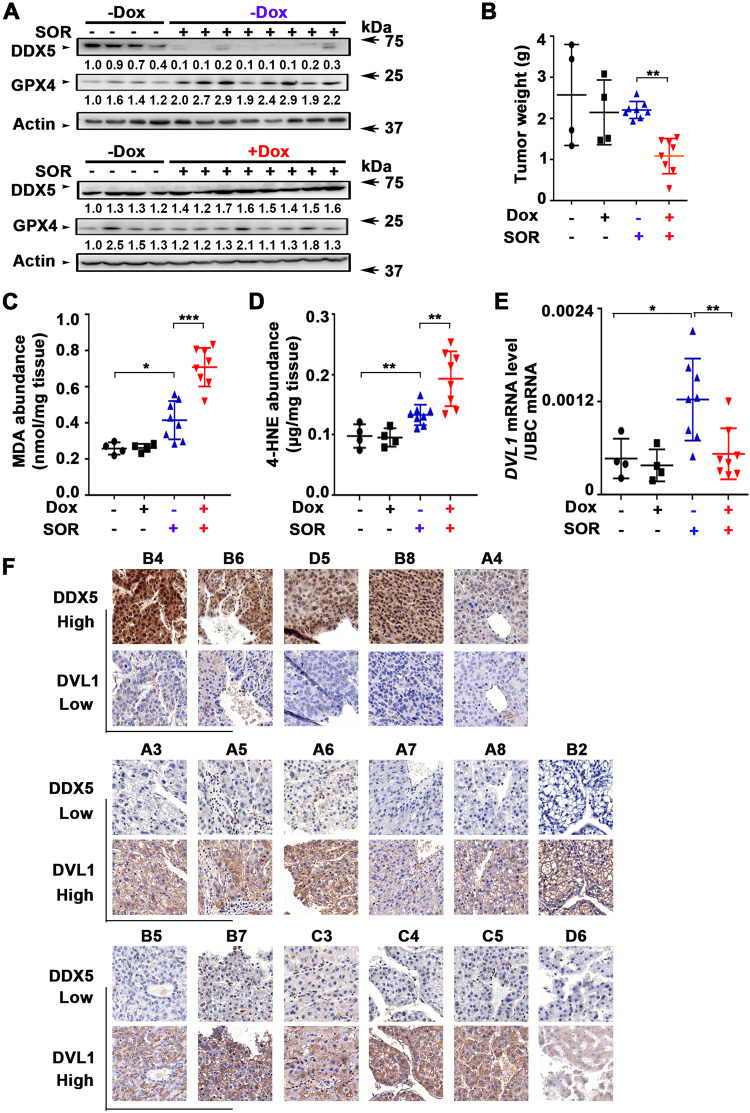
DDX5 overexpression enhances anti-tumor efficacy of sorafenib in Huh7 xenograft tumors. **A** DDX5 and GPX4 immunoblots of lysates from Dox-inducible Huh7-DDX5 tumors, ±Dox and SOR administration, as indicated (80 mg/kg, 5 days per week). Actin is used as a loading control. **B** Tumor weight for each treatment group from the indicated number of tumors. **p < 0.01 by unpaired *t-*test. Quantification of: **C** MDA (nmoles/mg tissue) and **D** 4-HNE (µg/mg tissue) using Huh7 xenograft tumors treated with ±DOX and SOR, as indicated. Data are expressed as mean ± SEM from eight tumors. *p < 0.05, ***p < 0.001 by unpaired *t-*test. **(E)** RT-PCR quantification of *DVL1* mRNA using total RNA isolated from Dox-inducible Huh7-DDX5 tumors, ±Dox and SOR, as indicated. Data expressed as mean ± SEM from the indicated number of tumors in each group. *p < 0.05, **p < 0.01 by unpaired *t-*test. **F** Immunohistochemistry (IHC) of indicated HCCs from TMA described in Fig. 1, with DDX5 and DVL1 antibodies (Numbers indicate tumor position in TMA, Fig. S8B). Representative images at 20× magnification.

Next, we examined whether DDX5 levels modulate sorafenib sensitivity in vivo using the Dox-inducible Huh7-DDX5 expressing cell line for xenograft tumor generation. Mice bearing Dox-inducible Huh7-DDX5 xenografts were fed or not with doxycycline-containing H_2_O starting 48 h prior to sorafenib administration for 10 days (Supplementary Fig. S8A). Xenograft tumors from animals treated with sorafenib without Dox administration exhibited nearly complete loss of endogenous DDX5, while GPX4 protein levels increased. By contrast, xenograft tumors from Dox-fed animals treated with sorafenib exhibited sustained DDX5 protein levels, absence of GPX4 induction (Fig. 8A), and reduced tumor weight in comparison to those without Dox administration (Fig. 8B and Supplementary Fig. S8B). Consistently, xenograft tumors from Dox-treated animals had significantly increased MDA and 4-HNE levels, indicative of ferroptosis in vivo (Fig. 8C, D). Notably, DDX5 overexpression ( + Dox) did not affect tumor growth in the absence of sorafenib (Fig. 8B and Supplementary Fig. S8B). In agreement with our in vitro results (Fig. 5E–H), in the absence of ectopic DDX5 expression (-Dox) sorafenib induced *DVL1* mRNA expression, whereas DDX5 overexpression (+Dox) abolished *DVL1* induction (Fig. 8E). In further support of this inverse relationship between DDX5 and DVL1 expression, we used the TMA employed in Fig. 1A, B and determined by IHC the expression of DVL1. HCCs exhibiting DDX5-positive immunostaining lacked DVL1 expression. By contrast, reduced DDX5 immunostaining is associated with positive DVL1 expression (Fig. 8F). These findings support our in vitro mechanistic results that DDX5 is an upstream negative regulator of DVL1 expression. We conclude that DDX5 determines the sorafenib response, via *DVL1* induction and Wnt/β-catenin pathway activation.

## Discussion

Herein, we provide clinical, in vitro, and in vivo evidence of the role of DDX5 in mTKI/sorafenib response. In clinical HCC samples reduced expression of DDX5 was associated with advanced tumor grade, and worst patient survival following treatment with sorafenib (Fig. 1). In liver cancer cell lines and preclinical HCC models sorafenib and mTKIs downregulate DDX5 (Figs. 2 and 3), and DDX5^KD^ cell lines exhibit reduced sensitivity to sorafenib [24]. Together, these observations suggested that DDX5 downregulation by sorafenib might be a contributing factor to sorafenib sensitivity. Here, we explored this hypothesis and identified its underlying mechanism.

### DDX5 promotes ferroptosis in sorafenib treated cells

Since sorafenib downregulates DDX5, we reasoned, that the viability of sorafenib-treated cells would be affected by DDX5 downregulation (siDDX5) or overexpression (DDX5^OE^). Indeed, siDDX5 increased whereas DDX5^OE^ significantly reduced cell viability in response to sorafenib (Fig. 4). Only the ferroptosis inhibitor ferrostatin rescued the effect of DDX5^OE^ on sorafenib-treated cells (Fig. 4), indicating DDX5 promotes ferroptosis. Using Dox-inducible cell lines that overexpress WT and ATPase-inactive DDX5, we found the RNA helicase activity of DDX5 is required for ferroptosis (Fig. S5), suggesting functionally active DDX5 represses expression of genes and pathways involved in ferroptosis.

### DDX5 downregulation activates Wnt/β-catenin signaling required for ferroptosis escape by sorafenib

The transcriptomic comparisons between WT and DDX5^KD^ cells treated ±sorafenib identified more than 300 genes mutually induced by sorafenib and repressed by DDX5 (Fig. 5). One of the top-ten predicted pathways associated with these upregulated, common genes is the Wnt pathway (Fig. 5), involved in every aspect of liver development [33] and HCC pathogenesis [46]. Upregulated Wnt signaling genes include among others *Wnt9A, Wnt7B,* and *DVL1* (Fig. 5). Recent studies link *Wnt9A* polymorphism to HCC risk [47], *Wnt7B* to sorafenib resistance [48], and *DVL1* to Wnt activation [45] and poor prognosis liver cancer [29]. Clinical data from TCGA also link *DVL1* overexpression to poor survival of HCC patients treated with sorafenib (Fig. 5). Importantly, the Huh7-DDX5^KO^ cell line conclusively demonstrates that the increased transcription of *DVL1* is DDX5-dependent, i.e., DDX5 is an upstream negative regulator of *DVL1* transcription, and in turn, of Wnt/β-catenin pathway activation (Fig. 6 and Supplementary Fig. S7). Together, our results show that overexpression of DDX5 induces ferroptosis (Fig. 4) and suppresses activation of Wnt/β-catenin signaling (Fig. 5). Conversely, DDX5 downregulation activates Wnt/β-catenin signaling required for enhanced cell viability and ferroptosis escape of HCC cells treated with sorafenib (Fig. 6). Interestingly, Wnt/β-catenin activation due to overexpression of Wnt receptor FZD10 was associated with levantinib resistance [49]; also, Wnt/β-catenin activation induced GPX4 expression and ferroptosis resistance in gastric cancer [36]. Our results show enhanced GPX4 expression and Wnt/β-catenin activation in DDX5^KO^ cells, as well as enhanced *GPX4* expression in HCCs with low *DDX5* mRNA (Fig. 4). Further studies are required to determine whether there is a link between GPX4 induction and activation of Wnt/β-catenin signaling by sorafenib in HCC.

How DDX5 represses transcription of many Wnt signaling genes, including *LRP5, Wnt7B, Wnt9a*, and *DVL1* among others, and how the RNA helicase activity of DDX5 regulates this process, is currently under investigation. Our preliminary data suggest that DDX5, as an RNA helicase, recruits a repressive epigenetic complex via interaction with a specific RNA. Since RNA hubs demarcate specific territories in the nucleus [50], we speculate that DDX5 recognizes and binds to specific RNAs/RNA secondary structures and recruits epigenetic effector complexes forming biomolecular condensates [51] that, in turn, modify the nearby chromatin.

### DDX5 overexpression enhances anti-tumor efficacy of sorafenib/mTKIs in vivo

Based on the mechanistic understanding of the role of DDX5 in sorafenib sensitivity presented herein, we identified two new approaches to improve the anti-tumor effectiveness of sorafenib/mTKIs. Firstly, Nanosac-encapsulated siRNA targeting *β-catenin* potentiates the anti-tumor activity of sorafenib in Huh7 xenografts (Fig. 7). The Nanosac-siRNA delivery approach served as proof-of-principle for demonstrating the significance of Wnt/β-catenin activation in the sorafenib response. Clinically, the use of lipid nanoparticles (LNPs) is a well-established approach to efficiently deliver siRNAs to hepatocytes [52]. Secondly, in sorafenib-treated Huh7 xenografts overexpression of DDX5 suppressed tumor growth and *DVL1* expression required for Wnt/β-catenin activation, inducing ferroptosis (Fig. 8). Thus, enhanced sorafenib anti-tumor efficacy is achieved either by inhibiting the Wnt/β-catenin pathway activated by DDX5 loss (Fig. 7) or by overexpression of DDX5 (Fig. 8). Moreover, human HCCs display an inverse relationship between low expression levels of DDX5 (Fig. 1A, B) and high expression of DVL1(Fig. 8F). High DVL1 levels are linked to poor prognosis HCC [29]. Also, the extrachromosomal circular miR17–92 amplicon [53], encoding the mir17-92 miRNA cluster that downregulates various tumor suppressors including DDX5 [24], was shown to be linked to poor prognosis HCC. Interestingly, nonalcoholic steatohepatitis (NASH), a condition leading to HCC, is also linked to the downregulation of *DDX5* mRNA levels by an unknown mechanism [54]. Given these independent studies that support the role of DDX5 deficiency in poor prognosis HCC [29, 53, 54], our observation that sorafenib targets the downregulation of DDX5 is novel. Specifically, it establishes a novel link between the progressive loss of DDX5 and the adaptive resistance mechanisms of HCCs to sorafenib. How sorafenib downregulates *DDX5* mRNA levels remains to be determined.

Since Wnt activation, in addition to ferroptosis escape by sorafenib, also regulates other oncogenic processes, including proliferation, survival, metabolism, immune tolerance, and angiogenesis [33], we propose that DDX5 overexpression in liver tumors could reverse or stall HCC progression [29], a promising therapeutic approach considering the recent success of RNA therapeutics [52, 55]. LNP-mediated siβ-catenin delivery or LNP-mediated overexpression of *DDX5* mRNA could be developed as novel therapies to suppress Wnt/β-catenin activation and DVL1 overexpression. Also, the recent identification of phytochemicals that enhance DDX5 protein stability [54] offers another feasible therapeutic strategy for improving the anti-tumor efficacy of sorafenib/mTKIs.

### Supplementary information

Supplementary Information

Original Data File

AJ checklist from OA

## Data Availability

All sequencing data are available from the NCBI Gene Expression Omnibus (GEO) database (accession number GSE199092).

## References

[1] El-Serag HB, Rudolph KL (2007). Hepatocellular carcinoma: epidemiology and molecular carcinogenesis. Gastroenterology.

[2] Llovet JM, Ricci S, Mazzaferro V, Hilgard P, Gane E, Blanc JF (2008). Sorafenib in advanced hepatocellular carcinoma. N Engl J Med.

[3] Kudo M, Finn RS, Qin S, Han KH, Ikeda K, Piscaglia F (2018). Lenvatinib versus sorafenib in first-line treatment of patients with unresectable hepatocellular carcinoma: a randomised phase 3 non-inferiority trial. Lancet.

[4] Bruix J, Qin S, Merle P, Granito A, Huang YH, Bodoky G (2017). Regorafenib for patients with hepatocellular carcinoma who progressed on sorafenib treatment (RESORCE): a randomised, double-blind, placebo-controlled, phase 3 trial. Lancet.

[5] Abou-Alfa GK, Meyer T, Cheng AL, El-Khoueiry AB, Rimassa L, Ryoo BY (2018). Cabozantinib in patients with advanced and progressing hepatocellular carcinoma. N Engl J Med.

[6] Zhu AX, Kang YK, Yen CJ, Finn RS, Galle PR, Llovet JM (2019). Ramucirumab after sorafenib in patients with advanced hepatocellular carcinoma and increased alpha-fetoprotein concentrations (REACH-2): a randomised, double-blind, placebo-controlled, phase 3 trial. Lancet Oncol.

[7] Finn RS, Qin S, Ikeda M, Galle PR, Ducreux M, Kim TY (2020). Atezolizumab plus bevacizumab in unresectable hepatocellular carcinoma. N Engl J Med.

[8] Galle PR, Finn RS, Qin S, Ikeda M, Zhu AX, Kim TY (2021). Patient-reported outcomes with atezolizumab plus bevacizumab versus sorafenib in patients with unresectable hepatocellular carcinoma (IMbrave150): an open-label, randomised, phase 3 trial. Lancet Oncol.

[9] Gordan JD, Kennedy EB, Abou-Alfa GK, Beg MS, Brower ST, Gade TP (2020). Systemic therapy for advanced hepatocellular carcinoma: ASCO guideline. J Clin Oncol.

[10] Zhu YJ, Zheng B, Wang HY, Chen L (2017). New knowledge of the mechanisms of sorafenib resistance in liver cancer. Acta Pharm Sin.

[11] Niu L, Liu L, Yang S, Ren J, Lai PBS, Chen GG (2017). New insights into sorafenib resistance in hepatocellular carcinoma: Responsible mechanisms and promising strategies. Biochim Biophys Acta Rev Cancer.

[12] Sun X, Ou Z, Chen R, Niu X, Chen D, Kang R (2016). Activation of the p62-Keap1-NRF2 pathway protects against ferroptosis in hepatocellular carcinoma cells. Hepatology.

[13] Gao R, Kalathur RKR, Coto-Llerena M, Ercan C, Buechel D, Shuang S (2021). YAP/TAZ and ATF4 drive resistance to Sorafenib in hepatocellular carcinoma by preventing ferroptosis. EMBO Mol Med.

[14] Stockwell BR, Friedmann Angeli JP, Bayir H, Bush AI, Conrad M, Dixon SJ (2017). Ferroptosis: a regulated cell death nexus linking metabolism, redox biology, and disease. Cell.

[15] Dai Z, Zhang W, Zhou L, Huang J (2023). Probing lipid peroxidation in ferroptosis: emphasizing the utilization of C11-BODIPY-based protocols. Methods Mol Biol.

[16] Dixon SJ, Stockwell BR (2019). The hallmarks of ferroptosis. Annu Rev Cancer Biol.

[17] Yang WS, SriRamaratnam R, Welsch ME, Shimada K, Skouta R, Viswanathan VS (2014). Regulation of Ferroptotic Cancer Cell Death by GPX4. Cell.

[18] Pereira B, Billaud M, Almeida R (2017). RNA-binding proteins in cancer: old players and new actors. Trends Cancer.

[19] Andrisani O, Liu Q, Kehn P, Leitner WW, Moon K, Vazquez-Maldonado N (2022). Biological functions of DEAD/DEAH-box RNA helicases in health and disease. Nat Immunol.

[20] Linder P, Jankowsky E (2011). From unwinding to clamping—the DEAD box RNA helicase family. Nat Rev Mol Cell Biol.

[21] Fuller-Pace FV (2013). The DEAD box proteins DDX5 (p68) and DDX17 (p72): multi-tasking transcriptional regulators. Biochim Biophys Acta.

[22] Zhang H, Xing Z, Mani SK, Bancel B, Durantel D, Zoulim F (2016). RNA helicase DEAD box protein 5 regulates Polycomb repressive complex 2/Hox transcript antisense intergenic RNA function in hepatitis B virus infection and hepatocarcinogenesis. Hepatology.

[23] Sun J, Wu G, Pastor F, Rahman N, Wang WH, Zhang Z (2022). RNA helicase DDX5 enables STAT1 mRNA translation and interferon signalling in hepatitis B virus replicating hepatocytes. Gut.

[24] Mani SKK, Yan B, Cui Z, Sun J, Utturkar S, Foca A (2020). Restoration of RNA helicase DDX5 suppresses hepatitis B virus (HBV) biosynthesis and Wnt signaling in HBV-related hepatocellular carcinoma. Theranostics.

[25] Lang X, Green MD, Wang W, Yu J, Choi JE, Jiang L (2019). Radiotherapy and immunotherapy promote tumoral lipid oxidation and ferroptosis via synergistic repression of SLC7A11. Cancer Discov.

[26] Friedmann Angeli JP, Krysko DV, Conrad M (2019). Ferroptosis at the crossroads of cancer-acquired drug resistance and immune evasion. Nat Rev Cancer.

[27] Yamashita T, Budhu A, Forgues M, Wang XW (2007). Activation of hepatic stem cell marker EpCAM by Wnt-beta-catenin signaling in hepatocellular carcinoma. Cancer Res.

[28] Toh TB, Lim JJ, Hooi L, Rashid M, Chow EK (2020). Targeting Jak/Stat pathway as a therapeutic strategy against SP/CD44+ tumorigenic cells in Akt/β-catenin-driven hepatocellular carcinoma. J Hepatol.

[29] Liao WY, Hsu CC, Chan TS, Yen CJ, Chen WY, Pan HW (2020). Dishevelled 1-regulated superpotent cancer stem cells mediate wnt heterogeneity and tumor progression in hepatocellular carcinoma. Stem Cell Rep.

[30] Sia D, Jiao Y, Martinez-Quetglas I, Kuchuk O, Villacorta-Martin C, Castro de Moura M (2017). Identification of an Immune-specific class of hepatocellular carcinoma, based on molecular features. Gastroenterology.

[31] Ruiz de Galarreta M, Bresnahan E, Molina-Sánchez P, Lindblad KE, Maier B, Sia D (2019). β-Catenin activation promotes immune escape and resistance to Anti-PD-1 therapy in hepatocellular carcinoma. Cancer Discov.

[32] Pinter M, Jain RK, Duda DG (2021). The current landscape of immune checkpoint blockade in hepatocellular carcinoma: a review. JAMA Oncol.

[33] Russell JO, Monga SP (2018). Wnt/β-catenin signaling in liver development, homeostasis, and pathobiology. Annu Rev Pathol.

[34] Liu T, Hu J, Han B, Tan S, Jia W, Xin Y (2021). A positive feedback loop of lncRNA-RMRP/ZNRF3 axis and Wnt/β-catenin signaling regulates the progression and temozolomide resistance in glioma. Cell Death Dis.

[35] Chen Y, Li X, Xu J, Xiao H, Tang C, Liang W (2022). Knockdown of nuclear receptor binding SET domain-containing protein 1 (NSD1) inhibits proliferation and facilitates apoptosis in paclitaxel-resistant breast cancer cells via inactivating the Wnt/β-catenin signaling pathway. Bioengineered.

[36] Wang Y, Zheng L, Shang W, Yang Z, Li T, Liu F (2022). Wnt/beta-catenin signaling confers ferroptosis resistance by targeting GPX4 in gastric cancer. Cell Death Differ.

[37] Ladner SK, Otto MJ, Barker CS, Zaifert K, Wang GH, Guo JT (1997). Inducible expression of human hepatitis B virus (HBV) in stably transfected hepatoblastoma cells: a novel system for screening potential inhibitors of HBV replication. Antimicrob Agents Chemother.

[38] Zhu X, Xu Y, Yu S, Lu L, Ding M, Cheng J (2014). An efficient genotyping method for genome-modified animals and human cells generated with CRISPR/Cas9 system. Sci Rep.

[39] Kim H, Yuk SA, Dieterly AM, Kwon S, Park J, Meng F (2021). Nanosac, a noncationic and soft polyphenol nanocapsule, enables systemic delivery of siRNA to solid tumors. ACS Nano.

[40] Subramanian A, Tamayo P, Mootha VK, Mukherjee S, Ebert BL, Gillette MA (2005). Gene set enrichment analysis: a knowledge-based approach for interpreting genome-wide expression profiles. Proc Natl Acad Sci USA.

[41] Terradillos O, Billet O, Renard CA, Levy R, Molina T, Briand P (1997). The hepatitis B virus X gene potentiates c-myc-induced liver oncogenesis in transgenic mice. Oncogene.

[42] Studach LL, Menne S, Cairo S, Buendia MA, Hullinger RL, Lefrançois L (2012). Subset of Suz12/PRC2 target genes is activated during hepatitis B virus replication and liver carcinogenesis associated with HBV X protein. Hepatology.

[43] Drummen GP, van Liebergen LC, Op den Kamp JA, Post JA (2002). C11-BODIPY(581/591), an oxidation-sensitive fluorescent lipid peroxidation probe: (micro)spectroscopic characterization and validation of methodology. Free Radic Biol Med.

[44] Labrecque CL, Fuglestad B (2021). Electrostatic drivers of GPx4 interactions with membrane, lipids, and DNA. Biochemistry.

[45] Ngo J, Hashimoto M, Hamada H, Wynshaw-Boris A (2020). Deletion of the Dishevelled family of genes disrupts anterior-posterior axis specification and selectively prevents mesoderm differentiation. Dev Biol.

[46] Tümen D, Heumann P, Gülow K, Demirci CN, Cosma LS, Müller M (2022). Pathogenesis and current treatment strategies of hepatocellular carcinoma. Biomedicines.

[47] Nahon P, Bamba-Funck J, Layese R, Trépo E, Zucman-Rossi J, Cagnot C (2023). Integrating genetic variants into clinical models for hepatocellular carcinoma risk stratification in cirrhosis. J Hepatol.

[48] Liu LJ, Lv Z, Xue X, Xing ZY, Zhu F (2022). Canonical WNT signaling activated by WNT7B contributes to L-HBs-mediated sorafenib resistance in hepatocellular carcinoma by inhibiting mitophagy. Cancers (Basel).

[49] Wang J, Yu H, Dong W, Zhang C, Hu M, Ma W (2023). N6-methyladenosine-mediated up-regulation of FZD10 regulates liver cancer stem cells’ properties and lenvatinib resistance through WNT/β-catenin and hippo signaling pathways. Gastroenterology.

[50] Quinodoz SA, Jachowicz JW, Bhat P, Ollikainen N, Banerjee AK, Goronzy IN (2021). RNA promotes the formation of spatial compartments in the nucleus. Cell.

[51] Sabari BR (2020). Biomolecular condensates and gene activation in development and disease. Dev Cell.

[52] Hoy SM (2018). Patisiran: first global approval. Drugs.

[53] Zou S, Chen S, Rao G, Zhang G, Ma M, Peng B, et al. Extrachromosomal circular MiR-17-92 amplicon promotes hepatocellular carcinoma. Hepatology. 2023. 10.1097/hep.0000000000000435.

[54] Zhang Y, Ye S, Lu W, Zhong J, Leng Y, Yang T (2023). RNA helicase DEAD-box protein 5 alleviates nonalcoholic steatohepatitis progression via tethering TSC complex and suppressing mTORC1 signaling. Hepatology.

[55] Woitok MM, Zoubek ME, Doleschel D, Bartneck M, Mohamed MR, Kießling F (2020). Lipid-encapsulated siRNA for hepatocyte-directed treatment of advanced liver disease. Cell Death Dis.

