# Supplementary material for: RNA helicase DDX5 modulates sorafenib sensitivity in hepatocellular carcinoma via the Wnt/β-catenin–ferroptosis axis
Source: Cell Death Dis. 2023 Nov 30;14(11):786. doi: 10.1038/s41419-023-06302-0 (PMC10689482; doi:10.1038/s41419-023-06302-0)
Supplement: Supplementary file 1 — Supplementary Information [file 41419_2023_6302_MOESM1_ESM.docx]

**Supplementary Information**

**RNA helicase DDX5 modulates sorafenib sensitivity in hepatocellular carcinoma via the Wnt/β-catenin-ferroptosis axis.**

Zhili Li^1,2^, Claude Caron de Fromentel^3^, Woojun Kim^2, 4^, Wen-Hung Wang^2^ , Jiazeng Sun^1,2^, Bingyu Yan^2, 5^, Sagar Utturkar^2^, Nadia Atalah Lanman^2, 6^, Bennett D. Elzey ^2,6^, Yoon Yeo^2, 4^, Hao Zhang^7^, Majid Kazemian^2, 5,8^, Massimo Levrero^3,9#^ and Ourania Andrisani^1,2*^

^1^Department of Basic Medical Sciences, Purdue University, ^2^Purdue Institute for Cancer Research, ^3^ Cancer Research Center of Lyon (CRCL) - INSERM U1052, CNRS5286, University Lyon, Université Claude Bernard Lyon 1, F69000 Lyon, France, ^4^Department of Industrial and Physical Pharmacy, Purdue University, ^5^Department of Biochemistry, Purdue University, ^6^Department of Comparative Pathobiology, Purdue University, ^7^Jiangsu Key Laboratory of Bioactive Natural Product Research and State Key Laboratory of Natural Medicines, School of Traditional Chinese Pharmacy, China Pharmaceutical University, Nanjing, China, ^8^Department of Computer Science, Purdue University, West Lafayette, IN 47907, USA, ^9^Hospices Civils de Lyon, Service d'Hépatologie et Gastroentérologie, Groupement Hospitalier Lyon Nord, France.

# co-Corresponding author: massimo.levrero@inserm.fr

*Corresponding author: andrisao@purdue.edu

Department of Basic Medical Sciences,

Purdue University

201 S. University Street

West Lafayette, IN  47907-2064

Phone: 765-494-8131

**Running title**: **DDX5 sensitizes liver cancer cells to sorafenib**

**Supplementary Figures**


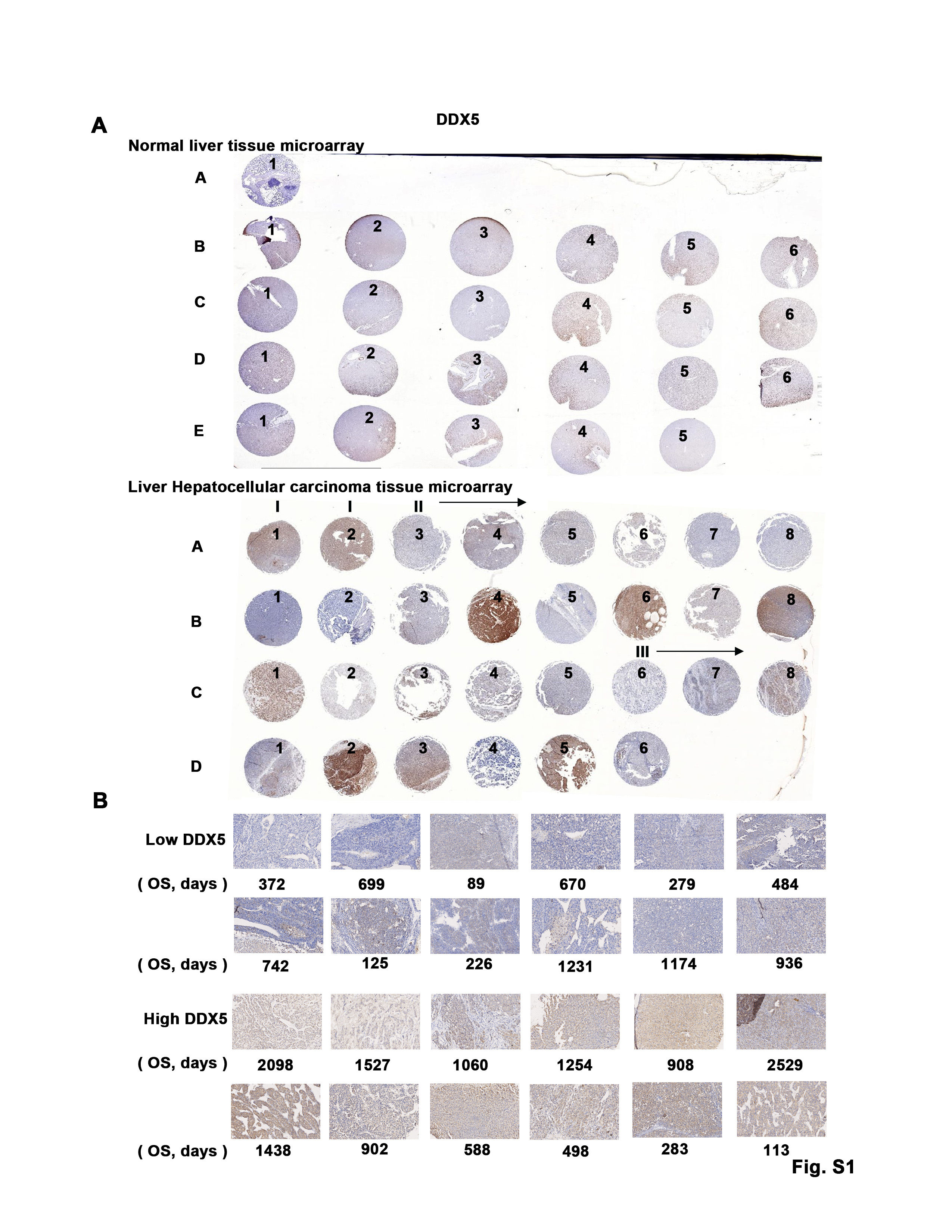


**Figure S1**. **(A)** Immunohistochemistry (IHC) with DDX5 antibody of normal liver tissue microarray (TMA) comprised of 24 samples and below of a TMA comprised of 30 human HCCs grades I-III. Images shown at 0.4X magnification. **(B)** IHC of DDX5 (10X magnification) of liver tumors from patients treated with sorafenib. Representative IHC images of 12 tumors from a total of 25 samples for each group are shown. Overall Survival (OS) following sorafenib treatment is indicated in days. Quantification is described in detail under Supplementary Materials and Methods.


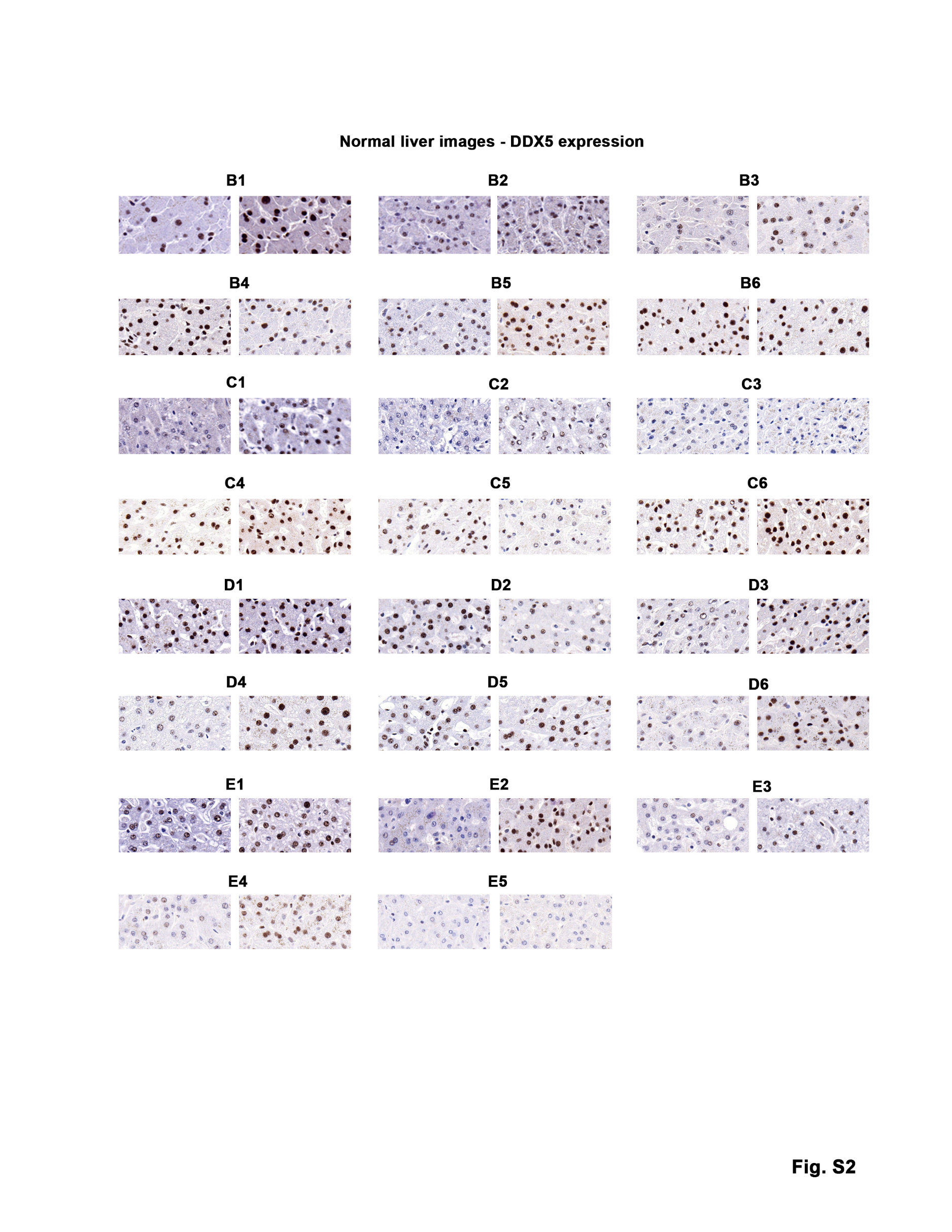
**Figure S2.** DDX5 IHC of normal liver TMA (from Fig. S1A top panel). Table S5 describes the clinical characteristics of each patient sample. Two fields at 20X magnification from, upper (left panel) and lower section (right panel) of each sample in TMA, is shown.


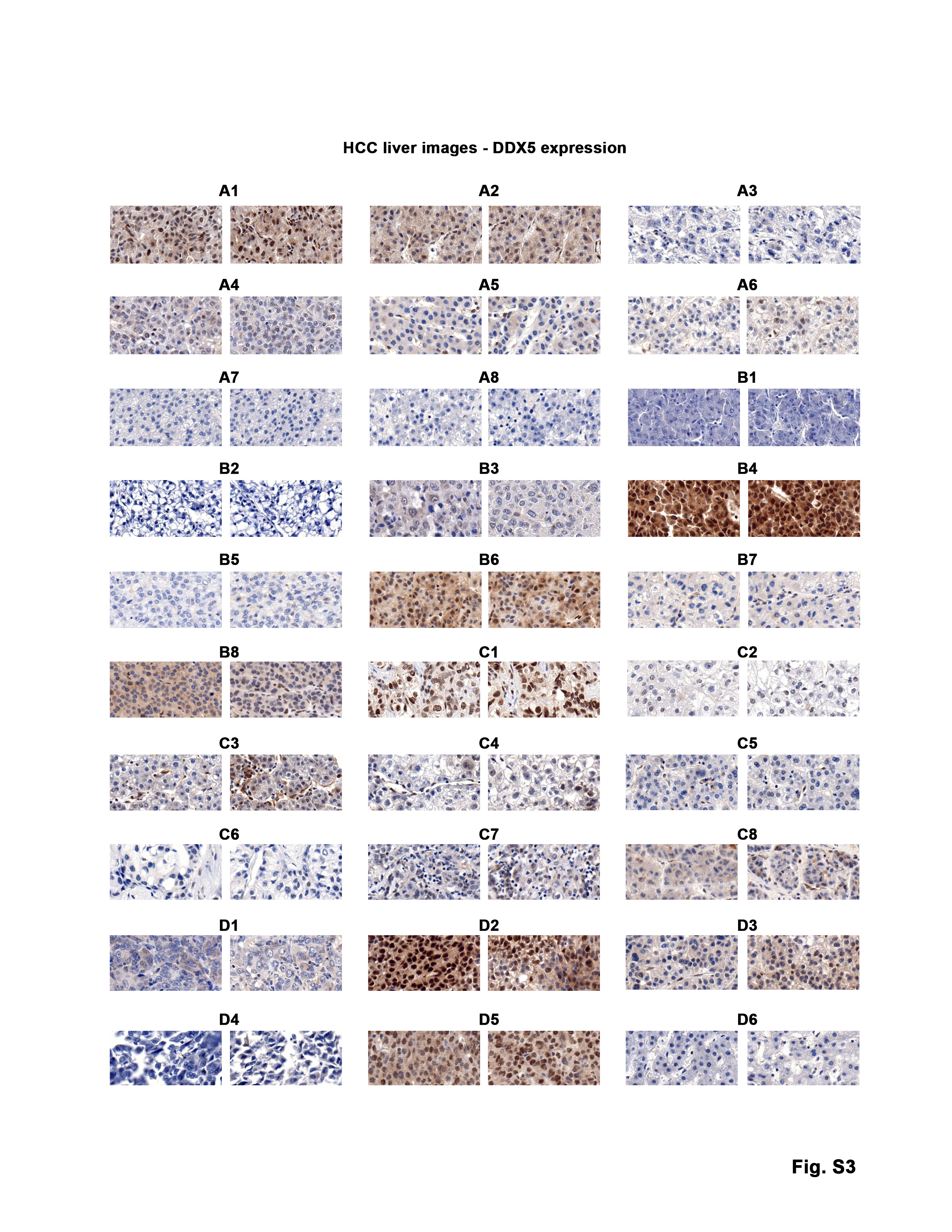


**Figure S3.** DDX5 IHC of TMA comprised of HCCs, grades I-III (from Fig. S1A lower panel). Two fields at 20X magnification from, upper (left panel) and lower section (right panel) of each sample in TMA, is shown.


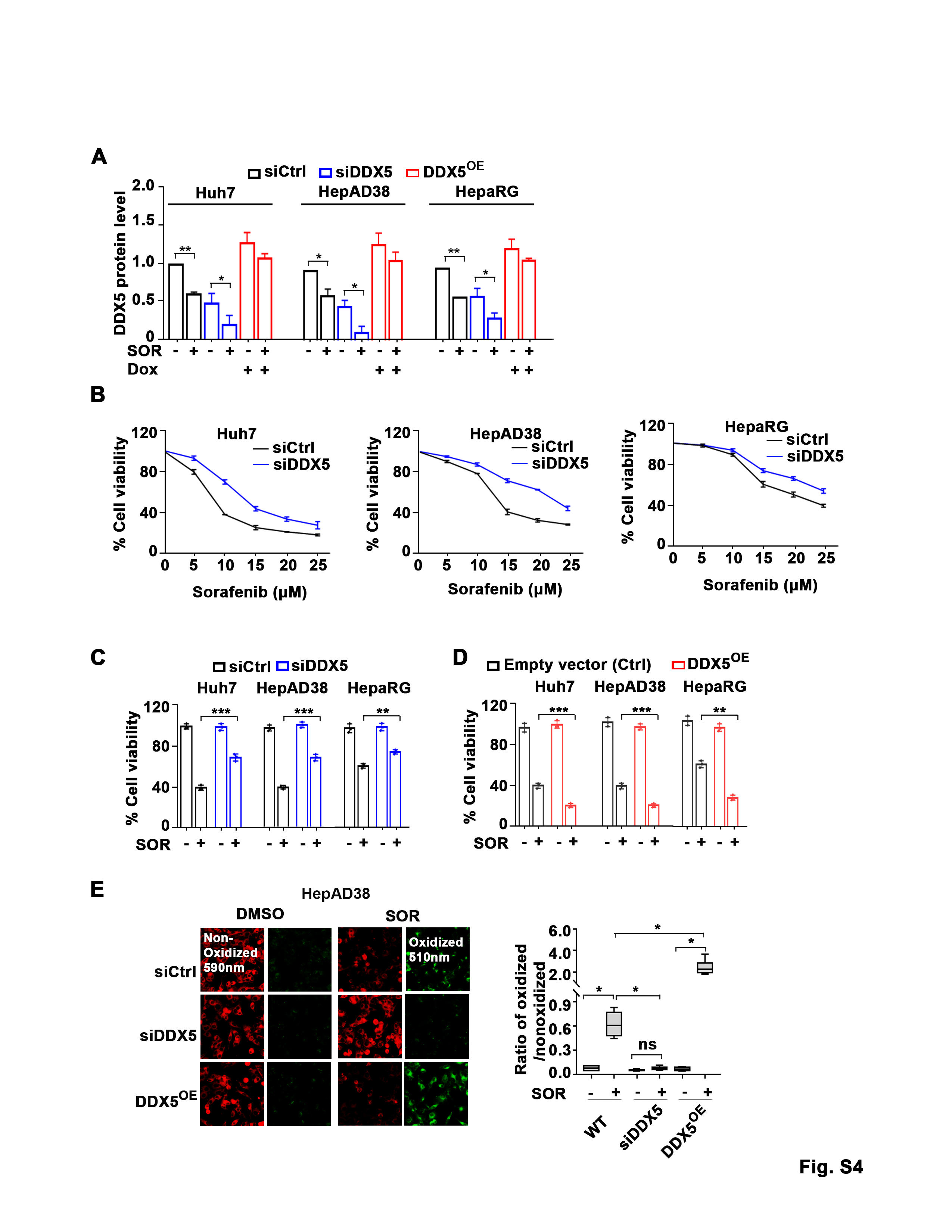
**Figure S4. (A)** Quantification of immunoblots of DDX5 in Huh7, HepAD38, and HepaRG cell lines transfected with siCtrl or siDDX5 for 24h, followed by addition for 24 h of SOR (10 µM for Huh7 and 15 µM for HepAD38 and HepaRG cell lines, using ImageJ software. For DDX5^OE^, indicated Dox-inducible-DDX5 cell lines were grown with Dox (1.0 µg/ml) for 48 h, and SOR for the last 24h. Error bars represent SD, n=3. *p<0.05, ** p<0.01 by unpaired *t-*test. Actin used as loading control. **(B)** Cell viability assays of Huh7, HepAD38 and HepaRG cell lines following siRNA mediated knockdown of DDX5 (sictrl vs. siDDX5 transfected), with increasing concentration of sorafenib. n=3**. (C-D)** Cell viability of Huh7, HepAD38, and HepaRG cells, transfected with indicated siRNAs for 24h, followed by addition of SOR for 24 h. For DDX5^OE^, indicated cell lines were grown with Dox (1.0µg/ml) for 48 h, and SOR for the last 24h. Data expressed as mean ± SEM, n=3. ** p<0.01, ***p<0.001 by unpaired *t-*test. Cell viability of Huh7. **(E)** Fluorescence microscopy of C11-BODIPY using HepAD38 cells, under conditions of siCtrl, siDDX5 or DDX5^OE^ as described in (Fig. S4A), treated +/- SOR (15 µM) for 24 h. Quantification by ImageJ software of ratio of oxidized (510 nm)/non-oxidized (590 nm) C11-BODIPY. Data are expressed as mean ± SEM from >1000 cells per condition. *p<0.05 by unpaired *t-*test.

**
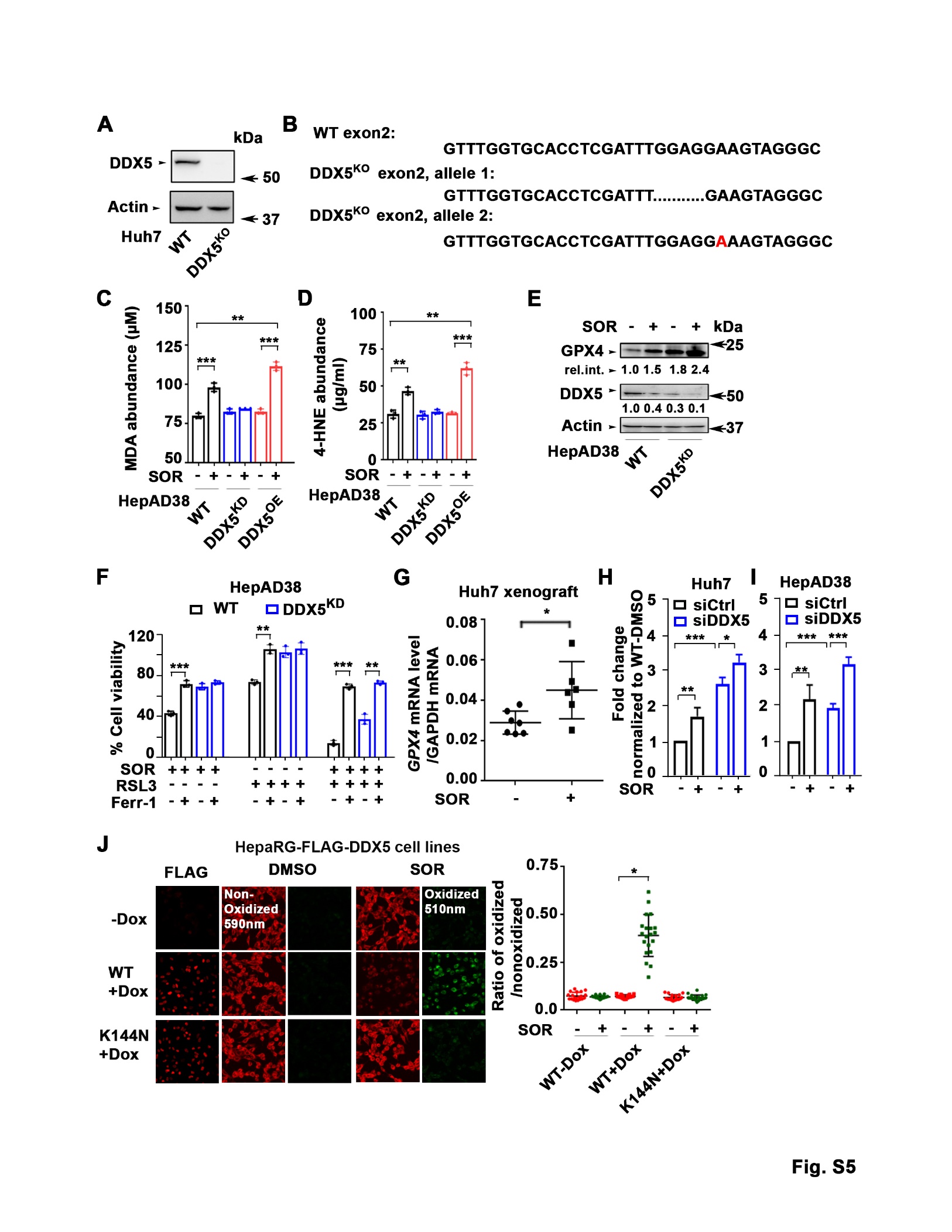
 Figure S5. (A)** Immunoblots of DDX5 in WT and DDX5^KO^ Huh7 cells. **(B).** Nucleotide sequence of WT DDX5 Exon2 and allele1 and 2 of DDX5^KO^. **(C)** MDA abundance (µM) and **(D)** 4-HNE abundance (µg/ml) quantified using lysates from HepAD38 wild type (WT), DDX5^KD^, and DDX5^OE^ cells treated without (-) or with (+) SOR for 24 hr. Data are expressed as SD, n=3. ** p<0.01, ***p< 0.001 by unpaired *t-*test. **(E)** Immunoblots of GPX4 and DDX5, as indicated, using lysates from WT and DDX5^KD^ HepAD38 cells grown without (-) or with (+) SOR for 24 hr. Relative intensity quantified vs. actin. A representative experiment is shown from n=3. **(F)** Cell viability of WT and DDX5^KD^ HepAD38 cells treated with SOR, RSL3 (0.5 µM) or Ferr-1 (10 µM), as indicated, for 24 h. Data expressed as mean ± SEM, n=3. ** p<0.01, ***p< 0.001 by unpaired *t-*test. **(G-I)** RT PCR quantification of *GPX4* mRNA using total RNA isolated from: **(G)**, Huh7 xenograft tumors from animals treated with (+) and without (-) sorafenib, described in Fig. 2A, (H) Huh7 and (I) HepAD38 cells transfected with siCtrl or siDDX5, as indicated, and treated +/-SOR for 24h. Data expressed as mean ± SEM, n=3. ** p<0.01, ***p< 0.001 by unpaired *t-*test. **(J)** Dox-inducible-DDX5-HepaRG cell lines treated +/- Dox for 24 h to express WT FLAG-DDX5 or inactive FLAG-K144N-DDX5. Immunofluorescence microscopy of FLAG-DDX5 (with anti-FLAG), and oxidized (510 nm) and non-oxidized (590 nm) C11-BODIPY in cells treated with DMSO or SOR (15 µM) for 24h. Quantification by ImageJ software of ratio of oxidized (510 nm)/non-oxidized (590 nm) C11-BODIPY. Data are expressed as mean ± SEM from >1000 cells. *p<0.05 by unpaired *t-*test.


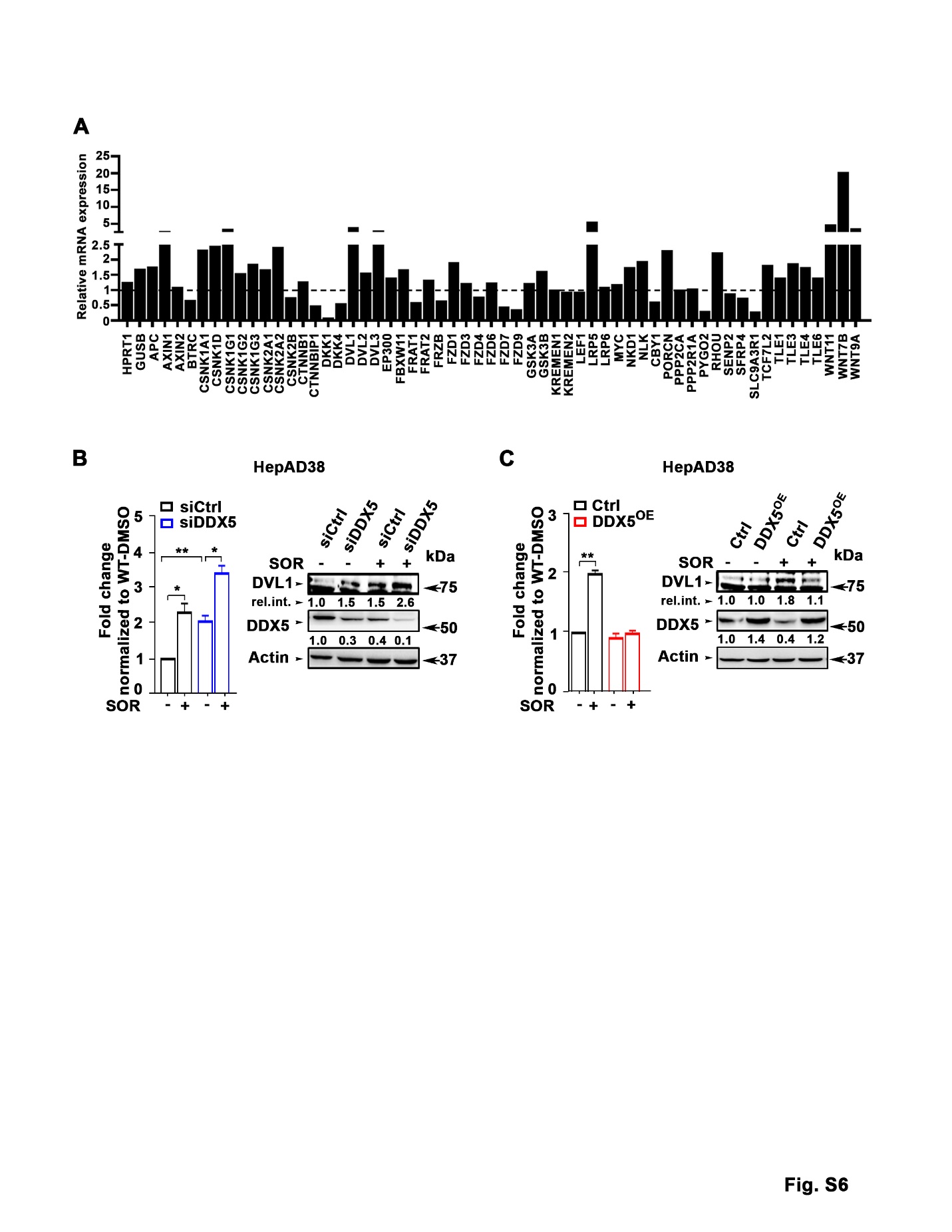


**Figure S6. (A)** Quantification of Wnt signaling genes using RNA from HepAD38 cells treated +/- SOR (7.5 μM for 3 days) and a 92 gene, Wnt signaling PCR microarray from Thermofisher. Results shown represent the average of two independent experiments. **(B-C)** qRT-PCR of *DVL1* mRNA and immunoblots of DVL1 protein, using total RNA or lysates, respectively, isolated from HepAD38 cells transfected with siCtlr or siDDX5 **(B),** and Dox-inducible HepAD38 DDX5^OE^ cells **(C)** treated +/- SOR (15 µM) for 24h. qRT-PCR data are expressed as mean ± SEM from three independent experiments. *p<0.05, **p<0.01 by unpaired *t-*test. A representative DVL1 immunoblot is shown from three independent experiments. Actin used as loading control.


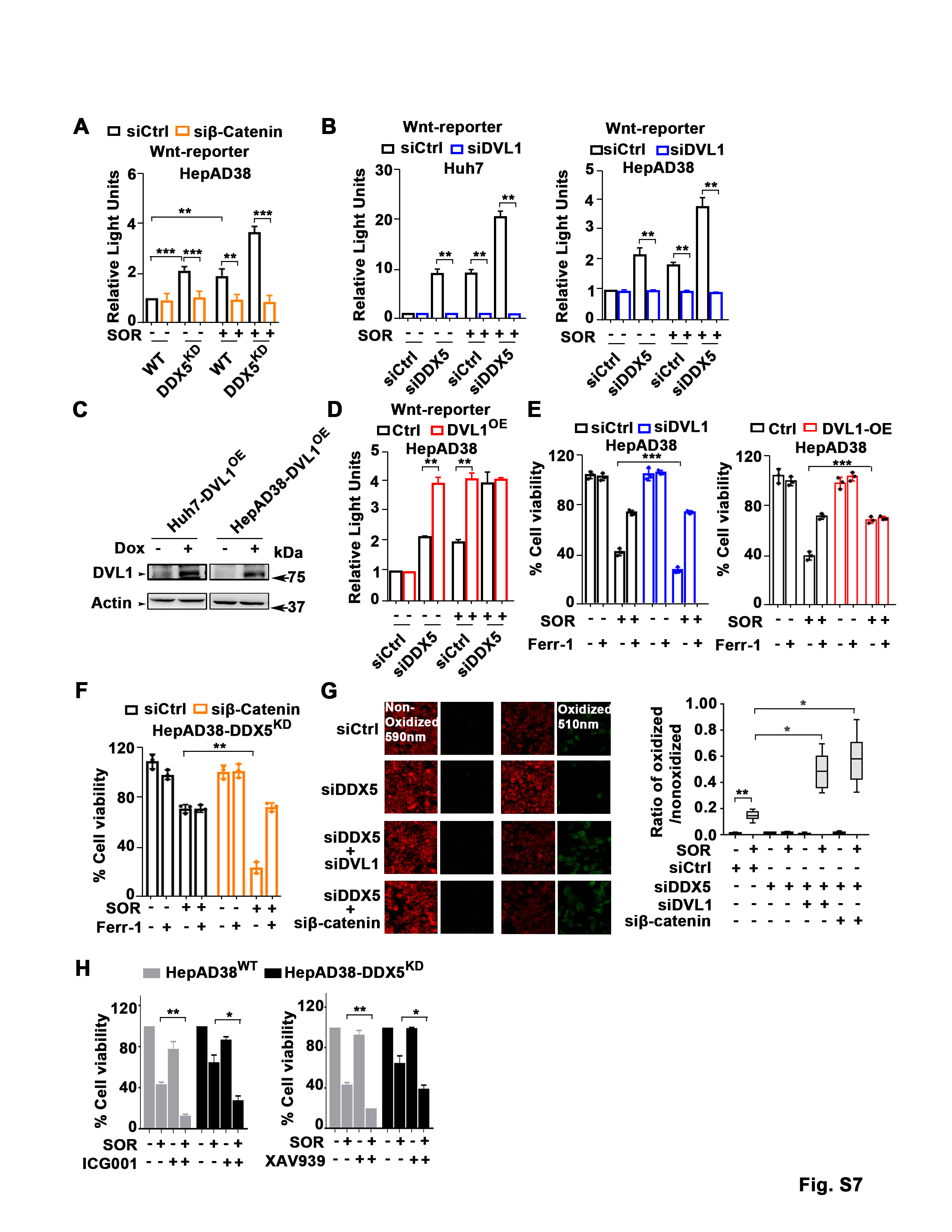
**Figure S7. (A)** Wnt-reporter (TopFlash) and Renilla-luciferase plasmids (100 ng each per 12-well plate) co-transfected in WT and DDX5^KD^ HepAD38 cells with siRNAs (50pM each) siCtrl, siβ-catenin, as indicated, treated +/- SOR (10 µM) for 24h. Data expressed as mean ± SEM, n=3. **p<0.01 ***p<0.001 by unpaired *t-*test**. (B)** Wnt-reporter (TopFlash) and Renilla-luciferase plasmids (100 ng each per 12-well plate) co-transfected in Huh7 and HepAD38 cells with siRNAs (50pM each) siCtrl, siDVL1, as indicated, treated +/- SOR (10 µM) for 24h. Data expressed as mean ± SEM, n=3. **p<0.01 ***p<0.001 by unpaired *t-*test. **(C)** Immunoblots of DVL1 in Dox-inducible Huh7-DVL1^OE^ and HepAD38-DVL1^OE^ cell lines, treated +/- Dox (1.0µg/ml) for 48hr. **(D)** Uninduced (Ctrl) and Dox-induced HepAD38-DVL1^OE^ cells co-transfected with Wnt-reporter (TopFlash) and Renilla-luciferase (100 ng each plasmid per 12-well plate), and siCtrl or siDDX5 as indicated, +/- SOR (15 µM) for 24h. Data expressed as mean ± SEM from n=3. *p<0.05, **p<0.01 by unpaired *t-*test. **(E)** Cell viability assays of HepAD38 cells with indicated siRNAs (siCtrl, siDVL1 )and HepAD38-DVL1^Ctrl^, HepAD38-DVL1^OE^ cells treated with SOR (10 µM), +/- Ferr-1 (10 µM) for 24 h. Data expressed as mean ± SEM, n=3. **p<0.01, ***p<0.001 by unpaired *t-*test. (F) Cell viability assays of HepAD38 cells with indicated siRNAs (siCtrl, si-β-catenin ) treated with SOR (10 µM), +/- Ferr-1 (10 µM) for 24 h. **(G)** Fluorescence microscopy of C11-BODIPY using HepAD38 cells transfected with siCtrl, siDDX5, siDVL1 or si-β-catenin treated +/- SOR (15 µM) for 24 h. Quantification by ImageJ software of ratio of oxidized (510 nm)/non-oxidized (590 nm) C11-BODIPY. Data are expressed as mean ± SEM from >1000 cells per condition. *p<0.05, ** p<0.01 by unpaired *t-*test. **(H**) Cell viability assays of indicated HepAD38 cells treated +/- SOR (10 µM), ICG001 (10 µM) and XAV393(20 µM), as indicated for 24 h. Data expressed as mean ± SEM, n=3. *p<0.05, **p<0.01 by unpaired *t-*test.


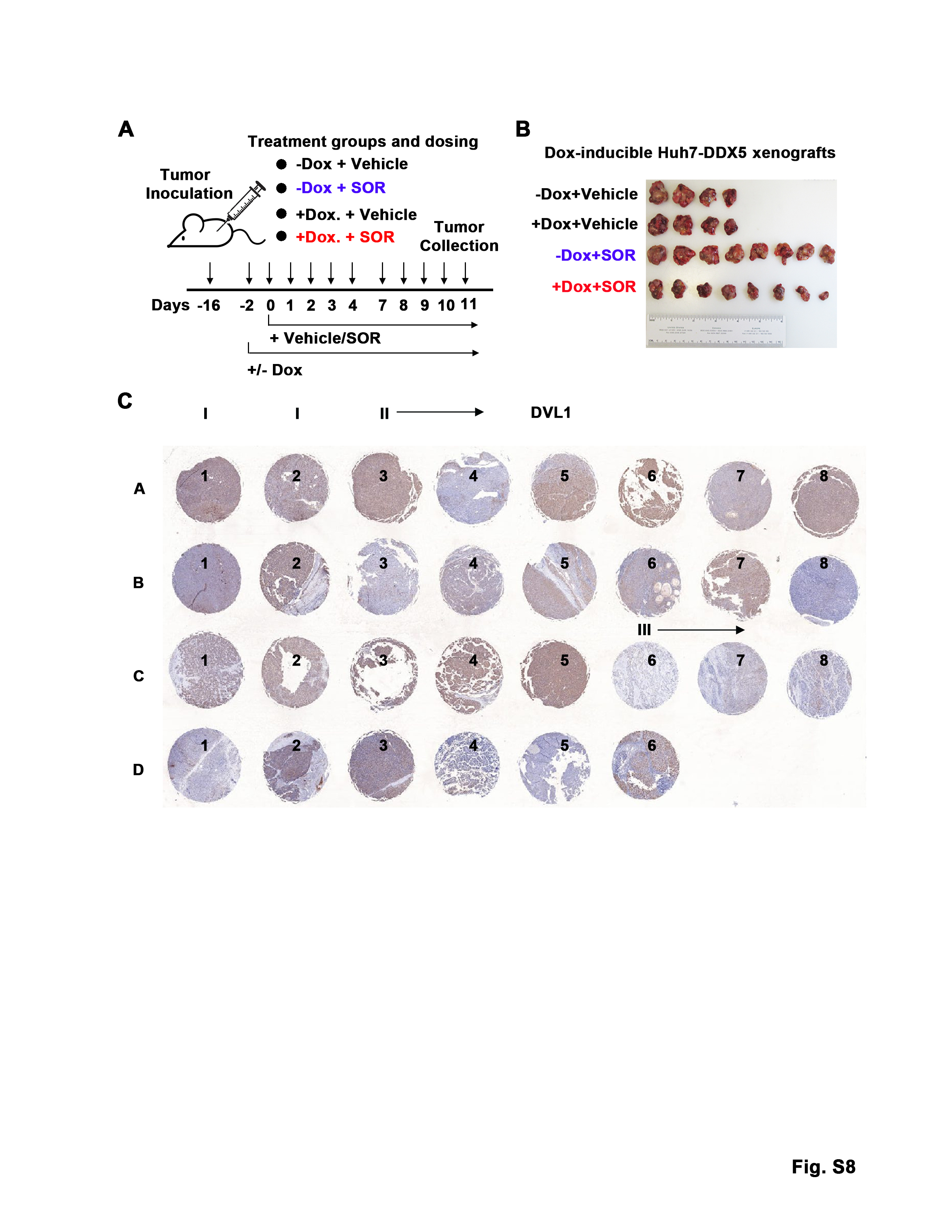


**Figure S8. (A)** Diagram illustrates treatment groups and timetable of Dox and SOR treatments.  **(B)** Images of Dox-inducible Huh7-DDX5 xenograft tumors, +/- Dox, collected on day12 of SOR or vehicle administration (Fig. 8). **(C)** Immunohistochemistry (IHC) with DVL1 antibody of a tissue microarray (TMA) comprised of 30 human HCCs grades I-III. Images shown at 0.4X magnification.

**Supplementary Materials and Methods**

**Quantification of Immunohistochemistry (IHC) of Fig. 1C and Fig. S1B :** Quantitative analysis of IHC Images of DDX5 staining were examined using a NanoZoomer 2.0 RS Pathology slide scanner (C10730-13, Hamamatsu) and NDP.view2 Image viewing software (U12388-01, HAMAMATSU). In order to improve the accuracy of statistical results, we randomly selected equal area fields of vision under 20X to calculate the proportion of DDX5-positive areas. Quantification of DDX5-positive regions was performed using 20X images and the Fiji program (Image J; National Institutes of Health). Customized evaluation protocols were optimized. In brief, digital images were imported as image sequence, followed by the spectral deconvolution method of DAB/hematoxylin color spectra for proper separation of the DAB color spectra. The threshold for positive staining was set and subsequently batch-processed to minimize technical variation or potential bias. The positive staining intensity was scored as proportion of DDX5-positive areas (Areas %), which sums all the pixels within the area and gives a total value within the threshold. DDX5 antibody：Abcam Catalog No. ab126730.

**Immunoblotting:** Cells were lysed (15 min, 4^o^C) in lysis buffer (Cell Signaling Technology), sonicated on ice for 30 s, and clarified by centrifugation (13,000 rpm, 15 min, 4^o^ C). Protein concentration was determined using BCA assay. All samples were diluted to 1µg/µL using 4x dye (Bio-Rad), and equal amounts of proteins (5.0- 40 μg per lane) were run on SDS-PAGE. Following electrophoresis, proteins were transferred to nitrocellulose membranes via wet transfer (200 mA, 45–90 min at 4^o^ C).SDS PAGE analyses of lysates used for immunoblotting utilized prestained MW markers (Precision Plus Protein Dual Color Standards, #1610374, from BIO-RAD ). Following transfer, nitrocellulose membranes were cropped according to migration of prestained MW markers. Membranes were blocked with 3% (w/v) BSA in Tris-buffered saline containing 0.1% (v/v) Tween 20 (TBST) and incubated with primary antibody in 3% (w/v) BSA in TBST for 1hr at room temperature, followed by incubation with secondary antibody (1:2000 dilution) in 3% BSA in TBST for 1h at room temperature. Three washes were performed after primary and secondary antibody incubation. Protein bands were detected by chemiluminescence using Pierce ECL (Bio-Rad). Densitometric analysis of immunoblots was performed using ImageJ software. Immunoblots are representative of three independent experiments. The antibodies used are listed in Supplementary Table S2.

**RNA preparation and qRT-PCR** RNA was isolated using the Purelink mRNA Mini kit (Life Technologies) or Direct Zol RNA miniprep kit (Zymo Research). cDNA was synthesized from 1.0 μg of total RNA using the iSCRIPT cDNA synthesis kit (Bio-Rad). qRT-PCR was performed using SYBR Green (Roche) in triplicate and normalized to GAPDH.

**Nanosac preparation:** Nanosacs carrying siCtrl and siβ-catenin were prepared as previously described (39). Nanosac-encapsulated siRNAs were administered every 48h intra-tumorally, delivering 3.0 µg siRNA per injection. Briefly, a sacrificial core of Nanosac, mesoporous silica nanoparticles (MSN), was prepared by adding a silica precursor, tetraethyl orthosilicate (TEOS), and -triethanolamine to micelles of cetyltrimethylammonium chloride (CTAC). Once the silica layers formed on the micelles, the surfactant was removed by washing the MSNs with methanol and HCl (500:19, v/v) for 48 h at room temperature. The formed MSNs were collected by centrifugation at 17000 relative centrifugal force (rcf) for 15 min, washed three times with methanol, and cationized by 3-aminopropyltriethoxysilane (APTES). The amine-modified MSN-APTES (MSN^a^) particles were collected by centrifugation at 17000 rcf for 15 min and washed thrice with ethanol. Purified MSN^a^ was mixed with siRNA (siCtrl or siβ-catenin) at a weight ratio of 150/1 (MSN^a^/siRNA) and incubated for 5 min at room temperature. The siRNA-loaded MSNs were incubated with dopamine hydrochloride solution in Tris buffer (10 mM, pH 8.5) for 24 h at room temperature to coat the polydopamine (pD) surface. After removing the remaining dopamine, the pD-coated siRNA-MSNs were mixed with an etch solution (0.36 M HF/1.44 M NH4F, pH 5) for 5 min to remove the sacrificial MSN core, washed three times with deionized water, and collected by centrifugation at 300 rcf for 10 min.

**Supplementary Table S1: List of Plasmids and siRNAs**

| **Plasmids, siRNAs** | **Source** |
| --- | --- |
| Renilla luciferase vector | Addgene (#27163) |
| TOPFlash vector | Addgene (#12456) |
| siCtrl | ThermoFisher Scientific (#4390843) |
| siDDX5-1 | ThermoFisher Scientific (#4392420, assay id s4007) |
| siDDX5-2 | ThermoFisher Scientific (#4392420, assay id s4008) |
| siDVL1 | ThermoFisher Scientific (# assay 14808) |
| siCTNNB1(catenin beta 1) | ThermoFisher Scientific (#146154, assay id AM16708) |

**Supplementary Table S2: List of Antibodies**

| **Antibody** | **Dilution** | **Application** | **Source** |
| --- | --- | --- | --- |
| Mouse α-Human DDX5 | 1:1000 in 2% BSA in TBST | Western Blot | Millipore Sigma (#05-850) |
| Rabbit α-Human DDX5 | 1:1000 in 2% BSA in TBST | Western Blot | Cell Signaling Technologies (#14994S) |
| Mouse α-Human Actin | 1:1000 in 2% BSA in TBST | Western Blot | Sigma (#A5441) |
| Horse α-Mouse secondary | 1:2000 in 2% BSA in TBST | Western Blot | Vector Laboratories (#PI-2000) |
| Goat α-Rabbit secondary | 1:2000 in 2% BSA in TBST | Western Blot | Vector Laboratories (#PI-1000) |
| Mouse FLAG | 1:1000 in 2% BSA in TBST | Western Blot | Sigma (#F1804) |
| DVL1 | 1:1000 in 2% BSA in TBST | Western Blot | ThermoFisher Scientific (#PA5-79176) |
| Rabbit α-DDX5 | 1/1000 | IHC | Abcam (#ab21696) |
| DVL1 | 1:200 | IHC | Abcam (#, ab233003) |
| Mouse α-Human GPX4 | 1:1000 | Western Blot | Proteintech  (#67763-1-Ig) |

**Supplementary Table S3: Primer sequences**

| **Primer** | **5’ – Sequence – 3’** |
| --- | --- |
| DDX5-F | AGCAAGTGAGCGACCTTATC |
| DDX5-R | CATCCTTCATGCCTCCTCTAC |
| GAPDH-F | CCCTTCATTGACCTCAACTACA |
| GAPDH-R | ATGACAAGCTTCCCGTTCTC |
| UBc-F | CCTGGAGGAGAAGAGGAAAGAGA |
| UBc-R | TTGAGGACCTCTGTGTATTTGTCA |
| DVL1-F | GCATAACCGACTCCACCATGTC |
| DVL1-R | GATGGAGCCAATGTAGATGCCG |
| β-catenin-F | CACAAGCAGAGTGCTGAAGGTG |
| β-catenin-R | GATTCCTGAGAGTCCAAAGACAG |
| mDDX5-F | ACGAATCTGTGGTCCTTTGG |
| mDDX5-R | CATAAACCACCAGCCATTCC |
| mGAPDH-F | GTCAAGGCCGAGAATGGGAA |
| mGAPDH-R | GCCTTCTCCATGGTGGTGAA |
| LRP5-F | GGACACCAACATGATCGAGTCG |
| LRP5-R | CGCTCAATGCTGTGCAGATTCC |
| WNT7B-F | AGAAGACCGTCTTCGGGCAAGA |
| WNT7B-R | AGTTGCTCAGGTTCCCTTGGCT |
| WNT9A-F | AGTGCCAGTTCCAGTTCCGCTT |
| WNT9A-R | AGGAGATGGCATAGAGGAAGGC |
| GPX4-F | ACAAGAACGGCTGCGTGGTGAA |
| GPX4-R | GCCACACACTTGTGGAGCTAGA |

**Supplementary Table S4: Reagents, Chemical inhibitors, and Kits**

| **Reagents, Chemical inhibitors, Kits** | **Source** |
| --- | --- |
| Sorafenib | Selleck Chemicals (#S7397) |
| Ferrostatin | Selleck Chemicals (#S7243) |
| Z-VAD-FMK | Selleck Chemicals (#S7023) |
| XAV-939 | Selleck Chemicals (#S1180) |
| ICG001 | Selleck Chemicals (#S2662) |
| Necrosulfonamide | Selleck Chemicals (#S8251) |
| Lenvatinib (E7080) | Selleck Chemicals (#S1164) |
| Regorafenib (BAY 73-4506) | Selleck Chemicals (#S1178) |
| CellTiter 96® AQueous One Solution Cell  Proliferation Assay (MTS) | Promega (#G3580) |
| PCR Mycoplasma Detection Kit | Applied Biological Materials (#G238) |
| Dual-Luciferase® Reporter Assay | Promega (#1980) |
| Cell Lysis Buffer (10X) | Cell Signaling Technology (#9803) |
| LightCycler® 480 SYBR Green I Master | Roche (#04887352001) |
| iScript™ cDNA Synthesis Kit | Biorad (#1708891) |
| Nitrocellulose Membrane, Roll, 0.2 µm | Biorad (#1620112) |
| LightCycler® 480 Sealing Foil | Roche (#04729757001) |
| LightCycler® 8-Tube Strips (white) | Roche (#06612601001) |
| DMSO | Sigma (#D8418-50ML) |
| Tween™ 20 | ThermoFisher Scientific (BP337-500) |
| Triton™ X-100 | Sigma (#T8787-100ML) |
| Tetracycline hydrochloride | Sigma (#T7660-5G) |
| Bovine Serum Albumin | Sigma (#A9647-100G) |
| Pierce™ ECL Western Blotting Substrate | ThermoFisher Scientific (#32106) |
| Geneticin™ Selective Antibiotic (G418 Sulfate) | ThermoFisher Scientific (#10131027) |
| Pierce™ BCA Protein Assay Kit | ThermoFisher Scientific (#23227) |
| Lipofectamine™ 3000 Transfection Reagent | ThermoFisher Scientific (#L3000015) |
| Lipofectamine™ RNAiMAX Transfection Reagent | ThermoFisher Scientific (#13778150) |
| Precision Plus Protein Dual Color Standards, | 500 µl #1610374, from BIO-RAD. |
| Restore™ PLUS Western Blot Stripping Buffer | ThermoFisher Scientific (#46430) |
| RNeasy Mini Kit | Qiagen (#74104) |
| Hoechst 33342 Solution (20 mM) | ThermoFisher Scientific (#62249) |
| Corning® 96 Well Black Polystyrene Microplate | Corning(#CLS3603-48EA) |
| CellTiter-Glo® 2.0 Cell Viability Assay | Promega( #G9242) |
| BODIPY™ 581/591 C11 (Lipid Peroxidation Sensor) | ThermoFisher Scientific ( # D3861) |
| 29 mm Glass bottom dish with 14 mm micro-well #1.5 cover glass | NC0662883（D29-14-1.5-N） |
| Nano-Glo® Luciferase Assay System | Promega( # N1110) |
| RNAlater™ Stabilization Solution | ThermoFisher Scientific ( # AM7024) |
| PEG400 | Selleck Chemicals (#S6705) |
| NE-PER™ Nuclear and Cytoplasmic Extraction Reagents | ThermoFisher Scientific ( # 78835) |
| Pierce™ 16% Formaldehyde (w/v), Methanol-free | ThermoFisher Scientific ( # 28908) |
| Protease Inhibitor Cocktail (100X) | Cell Signaling Technology (#5871) |
| NucRed™ Dead 647 ReadyProbes™ Reagent (TO-PRO-3 iodide) | ThermoFisher Scientific ( # R37113) |
| TaqMan™ Array Human WNT Pathway | Thermofisher (#4414100) |
| Malondialdehyde (MDA) lipid peroxidation Assay Kit | Abcam (ab233471) |
| Lipid Peroxidation (4-HNE) Assay Kit (96 TESTS) | Abcam (ab238538) |
| Liver Hepatocellular carcinoma tissue microarray | US Biolab Corporation  (HLivH030PG020) |
| Tissue Microarray - Normal 1910053 | Xenotech (TMA.NORM) |

**Supplementary Table S5: Tissue Microarray-Normal liver tissues, Donor information**

| Core | Donor | Pathology | Gender | Age  (Yrs) | Ethnicity | Macro  Fat(%) | BMI | Alcohol  Consumption |
| --- | --- | --- | --- | --- | --- | --- | --- | --- |
| A01 | Control | Uncharacterized | NA | NA | NA | NA | NA | NA |
| B01 | H0510 | Normal, Down syndrome | Male | 41 | African American | 0 | 24.8 | None |
| B02 | H0529 | Normal | Male | 41 | Caucasian | 0 | 24.5 | None |
| B03 | H0805 | Normal | Female | 24 | Arican American | 0 | 54.6 | None |
| B04 | H0881 | Normal, pulmonary fibrosis | Male | 48 | Caucasian | 1 | 27.3 | None |
| B05 | H0898 | Normal | Female | 58 | African American | 0 | 31.3 | None |
| B06 | H0919 | Normal | Female | 39 | Caucasian | <1 | 32.9 | Occasional |
| C01 | H1261 | Normal | Male | 43 | Caucasian | 0 | 20.1 | Occasional |
| C02 | H1263 | Normal | Male | 18 | Caucasian | 0 | 25.3 | None |
| C03 | H1265 | Normal | Male | 26 | Caucasian | 0 | 21.1 | Occasional |
| C04 | H1272 | Normal | Female | 20 | Caucasian | 0 | 21.2 | Occasional |
| C05 | H1279 | Normal | Male | 21 | Caucasian | 1 | 24.6 | None |
| C06 | H1285 | Normal, NIDDM | Male | 31 | Hispanic | 3 | 36.3 | Occasional |
| D01 | H1290 | Normal | Male | 51 | Hispanic | 0 | 30.2 | Occasional |
| D02 | H1291 | Normal | Male | 40 | Caucasian | <1 | 29.0 | Occasional |
| D03 | H1295 | Normal | Male | 45 | Caucasian | 0 | 29.8 | Occasional |
| D04 | H1307 | Normal, coronary artery disease | Male | 50 | Asian | <1 | 26.4 | None |
| D05 | H1314 | Normal | Male | 43 | Caucasian | 0 | 30.0 | None |
| D06 | H1318 | Normal | Male | 63 | Caucasian | 0 | 27.4 | None |
| E01 | H1321 | Normal | Female | 49 | Caucasian | <1 | 30.3 | None |
| E02 | H1330 | Normal, cerebral palsy | Female | 56 | Asian | 0 | 37.9 | None |
| E03 | H1338 | Normal, idiopathic lung disease | Female | 59 | Caucasian | 1 | 32.7 | Social |
| E04 | H1362 | Normal | Male | 56 | Caucasian | 0 | 13.7 | None |
| E05 | H1400 | Normal | Male | 41 | Caucasian | 0 | 25.7 | None |
