# Supplementary material for: RNA helicase DDX5 modulates sorafenib sensitivity in hepatocellular carcinoma via the Wnt/β-catenin–ferroptosis axis
Source: Cell Death Dis. 2023 Nov 30;14(11):786. doi: 10.1038/s41419-023-06302-0 (PMC10689482; doi:10.1038/s41419-023-06302-0)

Immunoblotting protocol is included under Supplementary Information section.

Please note: SDS PAGE analyses of lysates used for immunoblotting utilized prestained MW markers (Precision Plus Protein Dual Color Standards, #1610374, from BIO-RAD).

Following transfer, nitrocellulose membranes were cropped according to migration of prestained MW markers.

Fig 2A

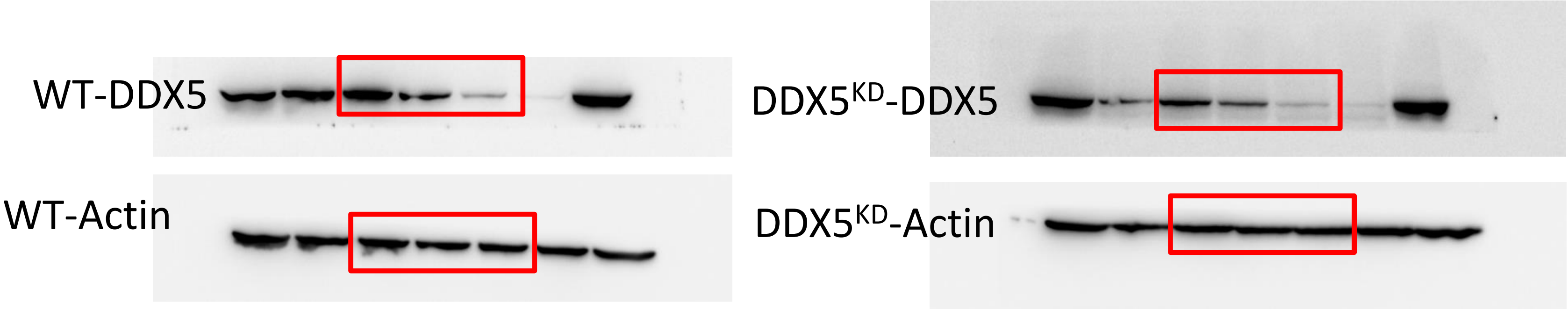
WT-DDX5
WT-Actin
DDX5KD-DDX5
DDX5KD-Actin

Fig 2E

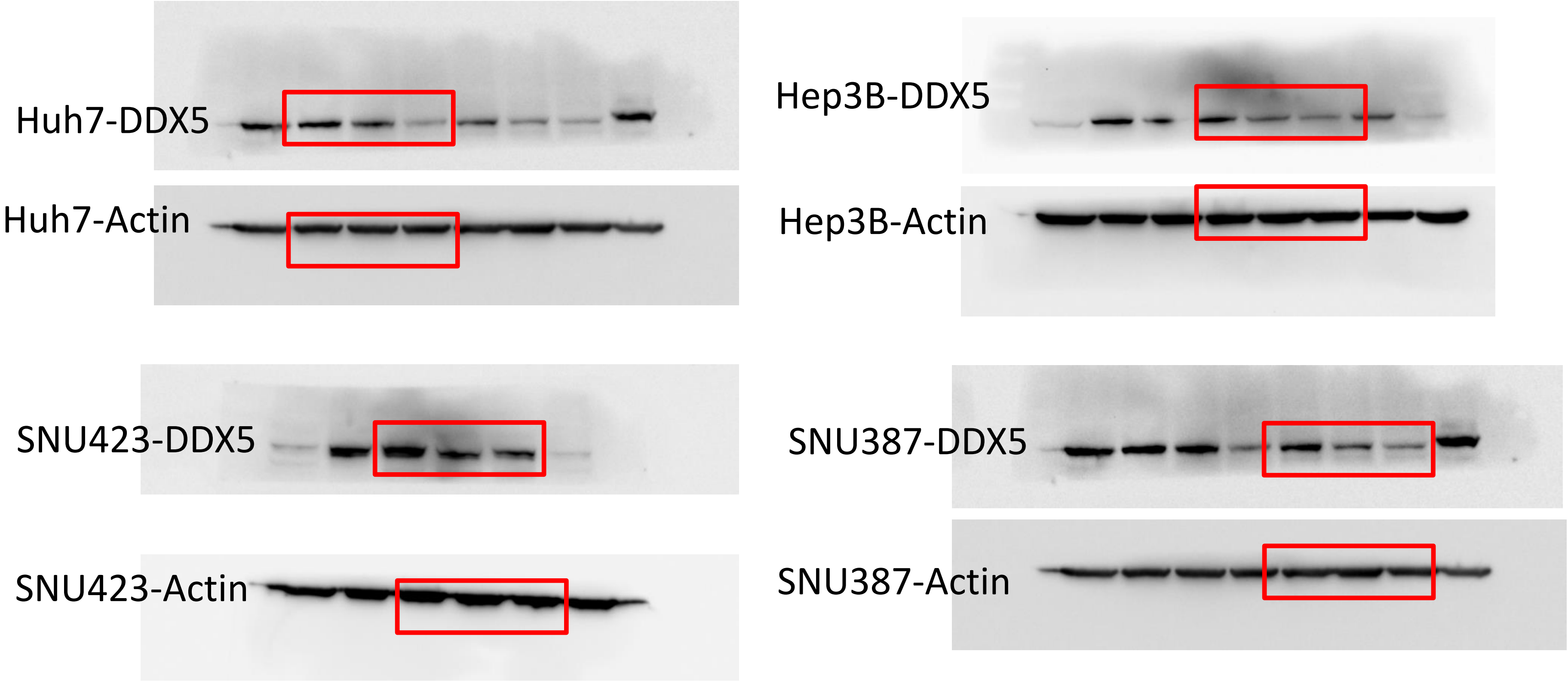
Huh7-DDX5
Huh7-Actin
Hep3B-DDX5
Hep3B-Actin
SNU423-DDX5
SNU423-Actin
SNU387-DDX5
SNU387-Actin

Fig 2F

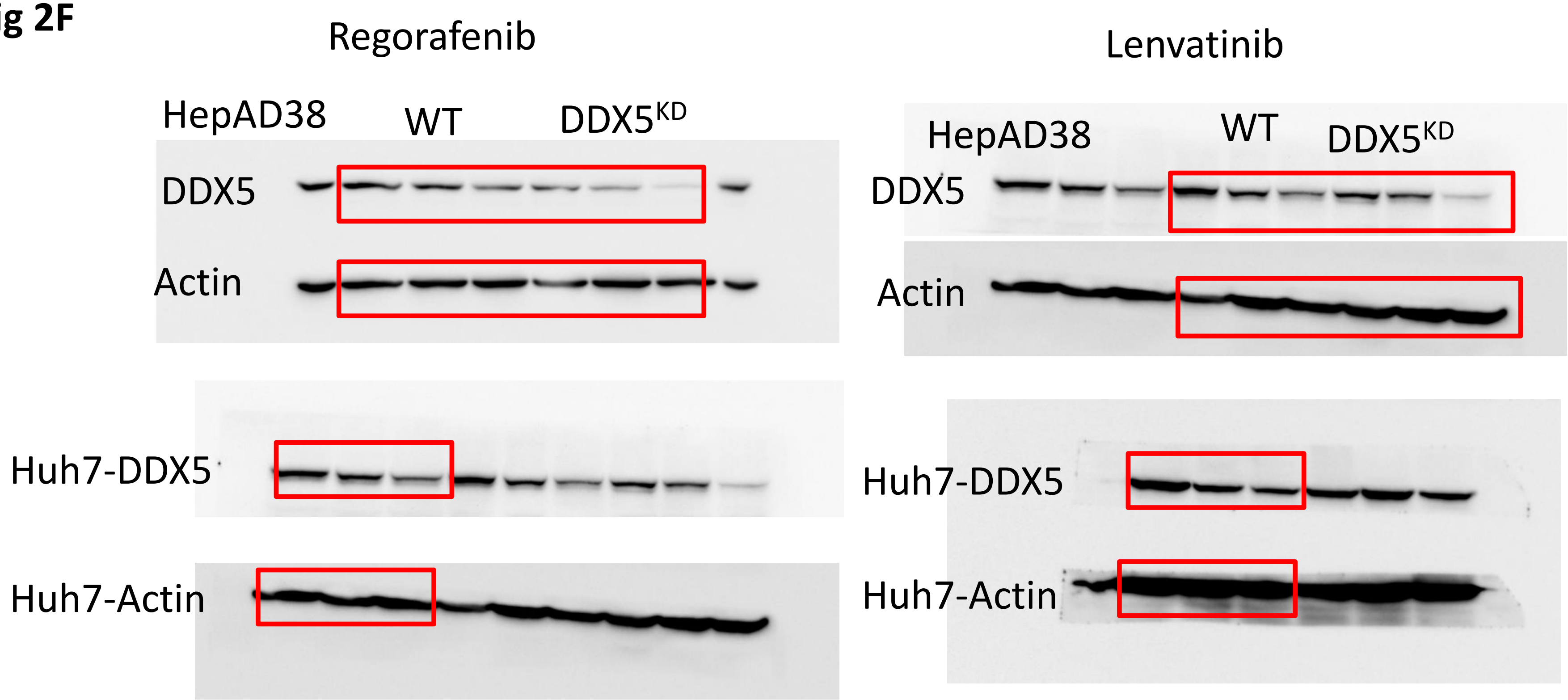
Regorafenib
Lenvatinib
HepAD38
WT
$DDX5^{KD}$
DDX5
Actin
HepAD38
WT
$DDX5^{KD}$
DDX5
Actin
Huh7-DDX5
Huh7-Actin
Huh7-DDX5
Huh7-Actin

Fig 3A

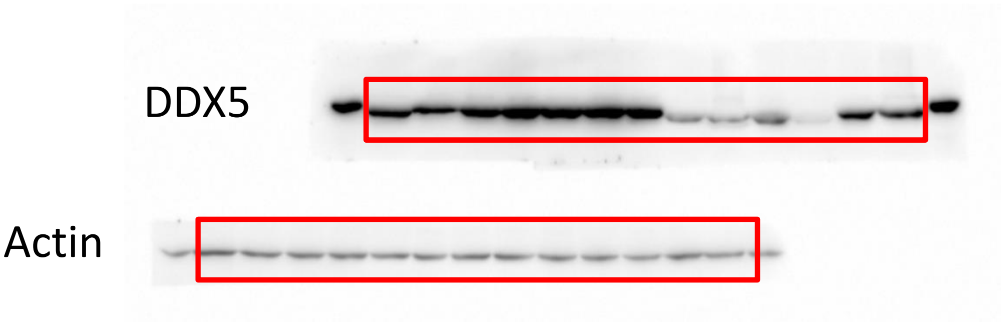
DDX5
Actin

Fig4A

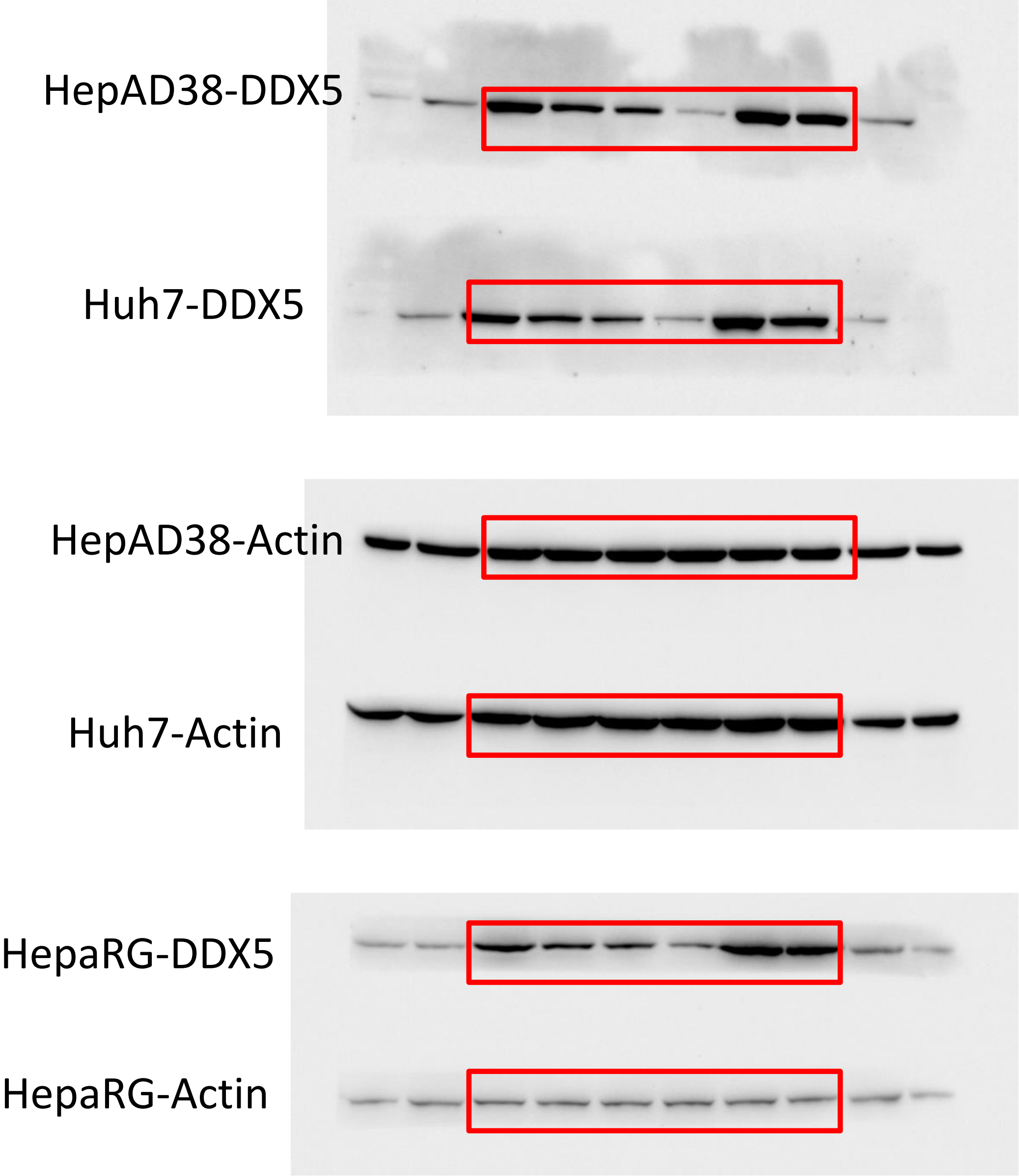
HepAD38-DDX5
Huh7-DDX5
HepAD38-Actin
Huh7-Actin
HepaRG-DDX5
HepaRG-Actin

Fig4F

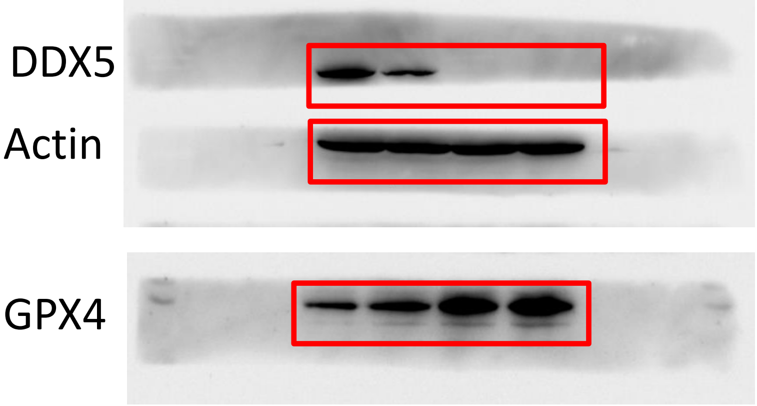
DDX5
Actin
GPX4

Fig5F

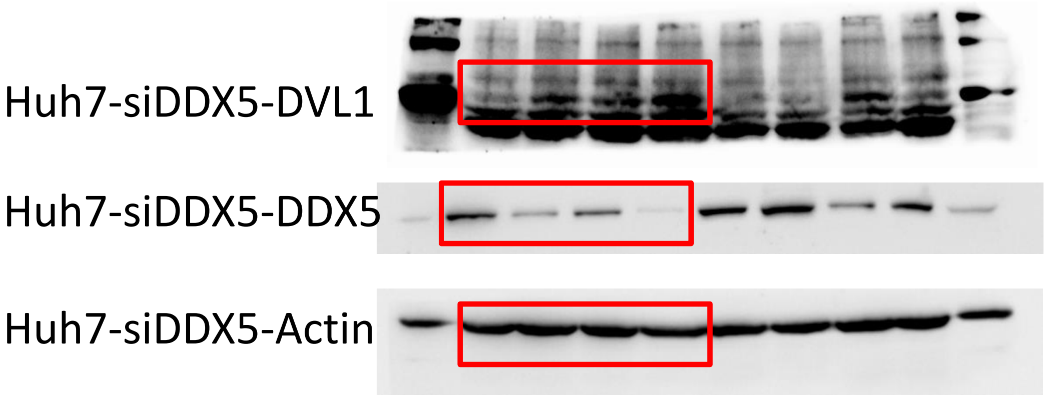

Fig5G

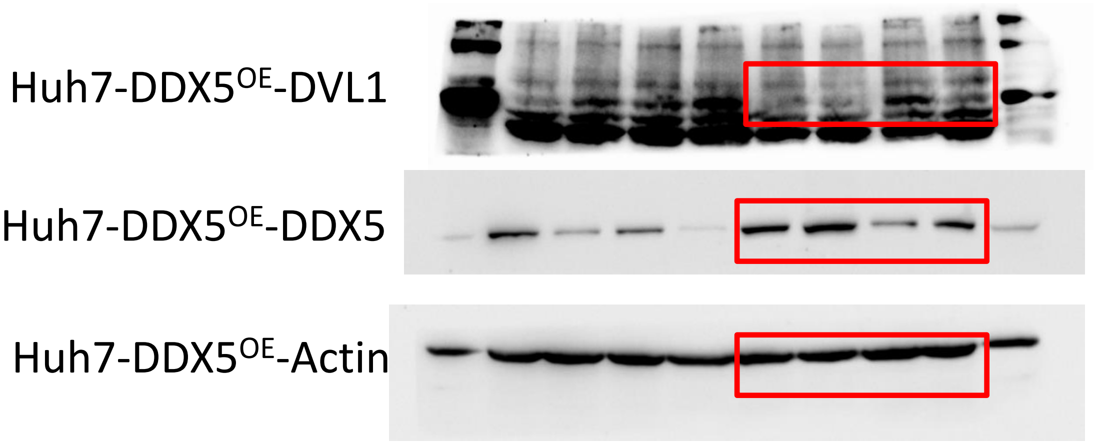

Fig5H

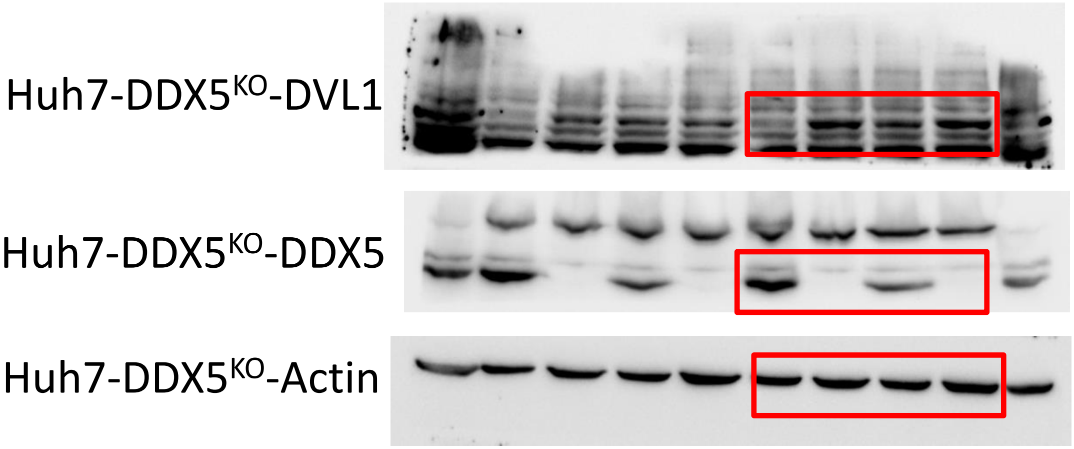

Fig8A

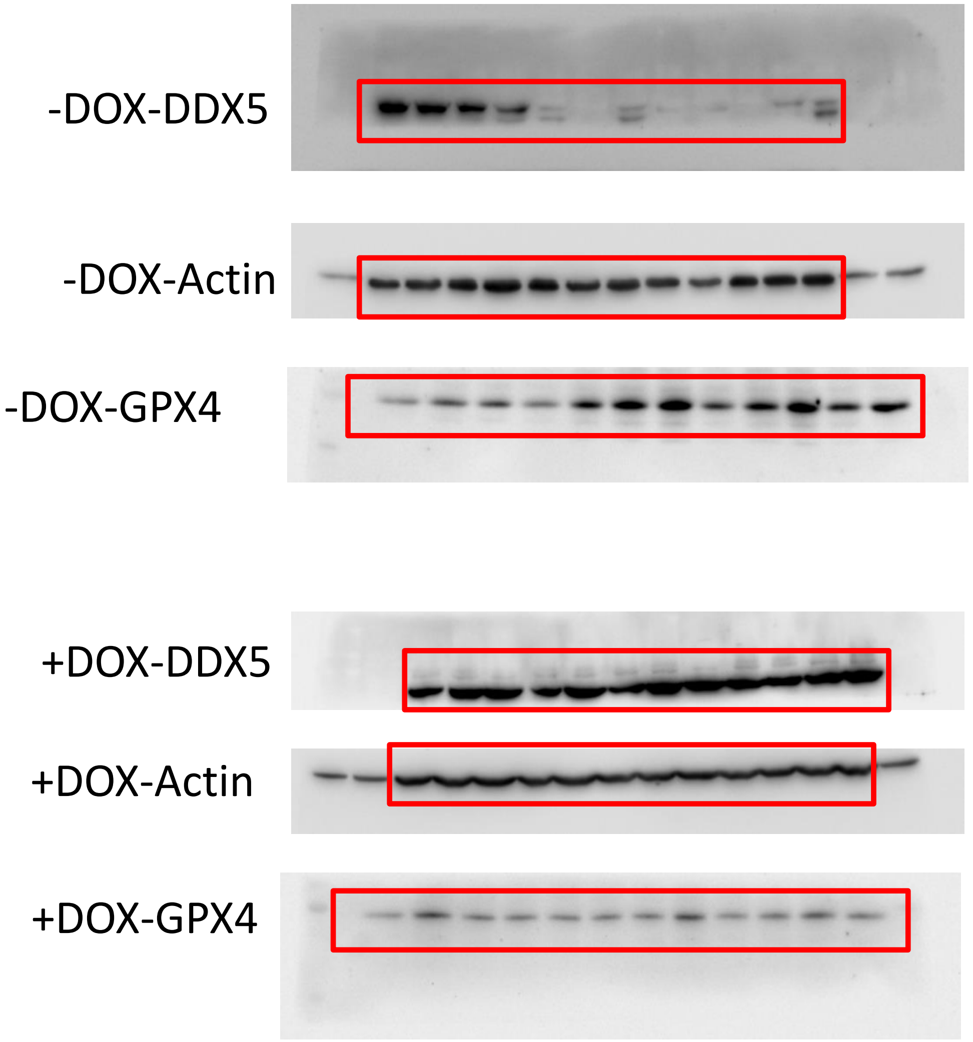

FigS5A

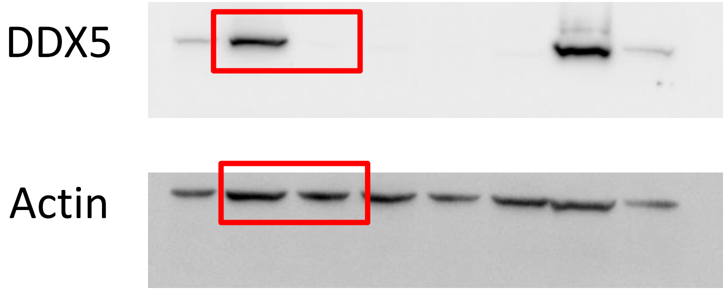

FigS5E

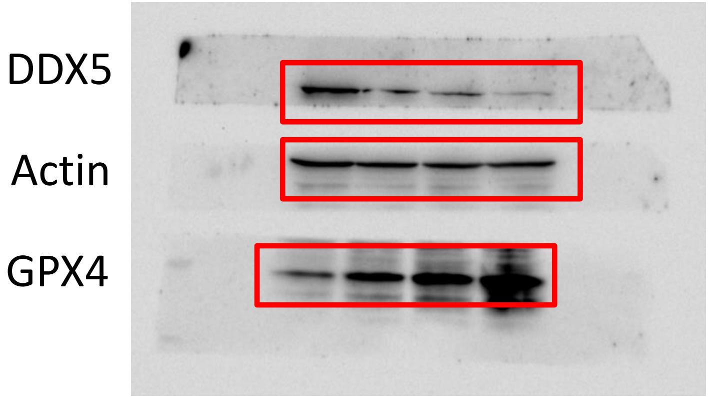

FigS6B

HepAD38-siDDX5-DVL1

HepAD38-siDDX5-DDX5

HepAD38-siDDX5-Actin

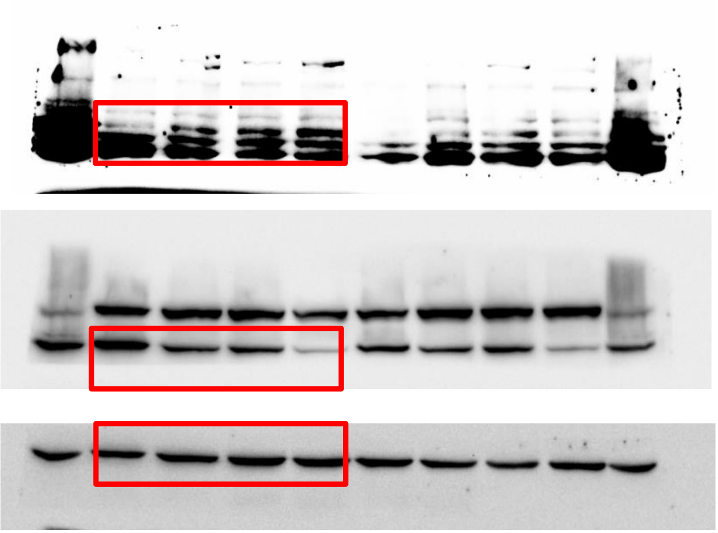

FigS6C

HepAD38-DDX5^{OE}-DVL1

HepAD38-DDX5^{OE}-DDX5

HepAD38-DDX5^{OE}-Actin

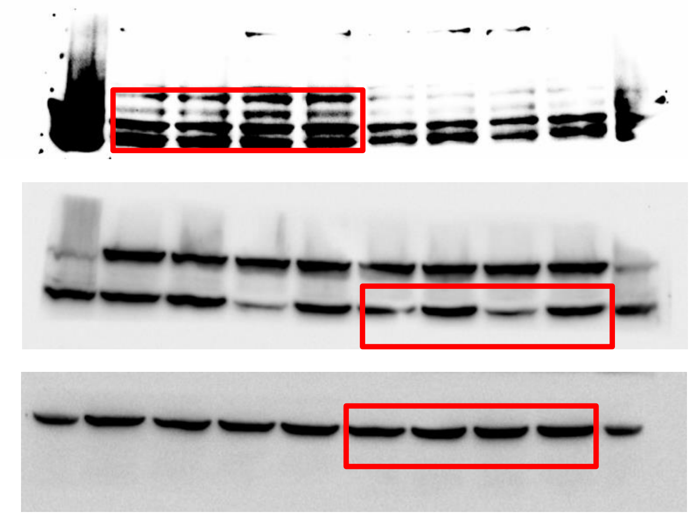

FigS7C

Huh7-DVL1^{OE}-DVL1

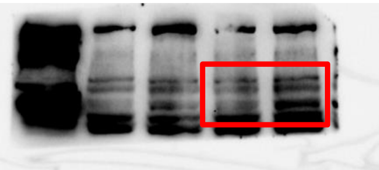

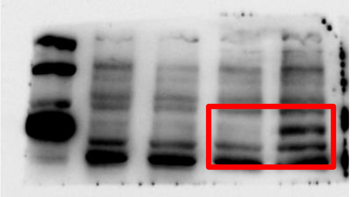

HepAD38-DVL1^{OE}-DVL1

Huh7-DVL1^{OE}-Actin

HepAD38-DVL1^{OE}-Actin

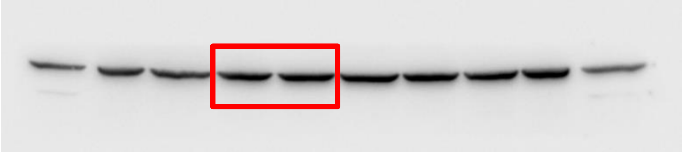

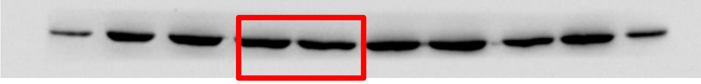

Supplement: Supplementary file 2 — Original Data File [file 41419_2023_6302_MOESM2_ESM.pdf]
